# MicroVQA: A Multimodal Reasoning Benchmark for Microscopy-Based Scientific Research

**Published:** 2025-03-17

**Authors:** James Burgess, Jeffrey J Nirschl, Laura Bravo-Sánchez, Alejandro Lozano, Sanket Rajan Gupte, Jesus G. Galaz-Montoya, Yuhui Zhang, Yuchang Su, Disha Bhowmik, Zachary Coman, Sarina M. Hasan, Alexandra Johannesson, William D. Leineweber, Malvika G Nair, Ridhi Yarlagadda, Connor Zuraski, Wah Chiu, Sarah Cohen, Jan N. Hansen, Manuel D Leonetti, Chad Liu, Emma Lundberg, Serena Yeung-Levy

**Affiliations:** 1 Stanford University; 2 Tsinghua University; 3 University of North Carolina at Chapel Hill; 4 Princeton University; 5 KTH Royal Institute of Technology; 6 Chan Zuckerberg Biohub Network

## Abstract

Scientific research demands sophisticated reasoning over multimodal data, a challenge especially prevalent in biology. Despite recent advances in multimodal large language models (MLLMs) for AI-assisted research, existing multimodal reasoning benchmarks only target up to college-level difficulty, while research-level benchmarks emphasize lower-level perception, falling short of the complex multimodal reasoning needed for scientific discovery. To bridge this gap, we introduce MicroVQA, a visual-question answering (VQA) benchmark designed to assess three reasoning capabilities vital in research workflows: expert image understanding, hypothesis generation, and experiment proposal. MicroVQA consists of 1,042 multiple-choice questions (MCQs) curated by biology experts across diverse microscopy modalities, ensuring VQA samples represent real scientific practice. In constructing the benchmark, we find that standard MCQ generation methods induce language shortcuts, motivating a new two-stage pipeline: an optimized LLM prompt structures question-answer pairs into MCQs; then, an agent-based ‘RefineBot’ updates them to remove shortcuts. Benchmarking on state-of-the-art MLLMs reveal a peak performance of 53%; models with smaller LLMs only slightly underperform top models, suggesting that language-based reasoning is less challenging than multimodal reasoning; and tuning with scientific articles enhances performance. Expert analysis of chain-of-thought responses shows that perception errors are the most frequent, followed by knowledge errors and then overgeneralization errors. These insights highlight the challenges in multimodal scientific reasoning, showing MicroVQA is a valuable resource advancing AI-driven biomedical research. MicroVQA is available here, project here.

## 1. Introduction

The transformative potential of AI in scientific discovery lies in its ability to interpret and reason over complex, multimodal data while integrating specialist domain knowledge. Recently, multimodal large language models (MLLMs) have demonstrated notable capabilities in data comprehension, recall, and multi-step reasoning across various modalities on exam-style questions [1, 3, 6, 49, 69, 81]. This progress has generated enthusiasm for scientific AI applications, ranging from multimodal chat AI assistants [33, 70, 85, 96] to AI agents capable of orchestrating complex experiments [12, 16, 26, 55]. However, while MLLMs perform well on general exam tasks, they often lack the specialized, contextual reasoning needed for real scientific challenges, where tasks require advanced image-based reasoning, analysis, and hypothesis-driven experimentation.

In recent years, efforts to apply scientific MLLMs across a range of domains have laid an essential groundwork. Advances have been achieved in image understanding [41, 43, 47, 50, 57], literature analysis [41, 47, 66, 77], and problem-solving at school and early college levels [53, 87]. In applied settings, AI has facilitated scientific tasks in biology [28, 60, 63, 68], chemistry [12, 38, 55], software [63, 73], and diagnostic pathology and radiology [52]. However, there has been less progress on multimodal tasks requiring *reasoning* in *research-level* settings. The gap is due in part to a lack of multimodal benchmarks for scientific reasoning – unlike college-level tasks where existing exams can form benchmarks, research tasks require expert curation.

Microscopy provides a compelling use case for developing such a benchmark. It requires more than pattern recognition or factual recall – it demands the ability to synthesize visual information with experimental context, formulate hypotheses, and propose experimental follow-ups. To evaluate these advanced capabilities, we introduce MicroVQA, a visual-question answering (VQA) benchmark tailored for multimodal scientific reasoning. Grounded in the real-world scientific practice of biological microscopy, MicroVQA incorporates 1,042 multiple-choice questions (MCQs) manually created by biological researchers across diverse disciplines, with each question taking more than 30 minutes to generate. Careful expert curation ensures the benchmark is relevant to real scientific applications.

MicroVQA is designed to evaluate three key reasoning tasks crucial to scientific inquiry: (1) *expert image understanding* – the ability to recognize scientifically relevant features and integrate contextual information about sample preparation; (2) *hypothesis generation* – the formulation of scientifically plausible explanations from experimental data; and (3) *experiment proposal* – the ability to suggest further experiments that could validate or refute hypotheses. These capabilities form a common workflow in scientific experimentation. They demand both abductive reasoning – inferring the best explanation from multiple possibilities – and deductive reasoning – moving from general principles to specific conclusions [81].

We tested standard methods to map open scientific VQA samples to multiple-choice questions (MCQs), but found that they induce language shortcuts – the MCQs failed to truly test multimodal abilities. To ensure question quality and difficulty, we developed a two-stage pipeline for MCQ generation: first, an optimized LLM prompt structures QA pairs into well-formatted MCQs; then, a novel agent-based ‘RefineBot’ system increases question difficulty by rewriting questions without language shortcuts. This ensures that MicroVQA’s MCQs better test true scientific capabilities, rather than simple pattern recognition.

We benchmarked frontier MLLMs on MicroVQA, finding that the best performers achieved a modest peak of 53%, which shows a significant gap between current models and expert-level scientific reasoning. The variation between different MLLMs was small; notably, smaller LLMs only slightly underperform their larger counterparts, suggesting that the key challenge for our MCQs is not language-only reasoning, but multimodal reasoning or knowledge. We also find that finetuning MLLMs on scientific publications improves MicroVQA performance. Finally, a group of biomedical researchers performed a detailed qualitative analysis into MLLM failure modes, uncovering three major failure modes: Expert perception is the most common, followed by knowledge errors, and then overgeneralization reasoning errors. This further supports that visual reasoning is a key challenge in MicroVQA.

In summary, our contributions are as follows:

- We introduce MicroVQA, a benchmark for multimodal reasoning in biological microscopy. We defined three key tasks for scientific research – expert image understanding, hypothesis generation, and experiment proposal. Then, expert researchers curated 1,042 diverse VQA samples.
- We develop a novel two-stage MCQ generation pipeline to address the challenge of creating MCQs from long-format scientific questions without language shortcuts.
- We provide quantitative and qualitative benchmarking of frontier MLLMs, highlighting areas for improvement.

## 2. Related work

### MLLM reasoning benchmarks

While *perception* focuses on identifying an image’s visual features, *visual reasoning* often integrates prior knowledge to derive new conclusions using logical inference strategies like deduction, induction, abduction, and analogical reasoning [81]. Reasoning in MLLMs has focused on image-text VQA [25, 44, 53, 81, 87, 88]. The most prominent examples in science are MMMU [87, 88], ScienceQA, [53] and Math-Vista [54]. While they do test reasoning, they are derived from exams up to the college level, while our benchmark emphasizes research-level difficulty. Other popular multimodal benchmarks test commonsense inference [71, 90], spatial or relational awareness [35, 91] and integrating prior knowledge [56]. Another line of work is visual abductive reasoning [8, 32], or finding the most likely explanation for an observation from incomplete information; this relates closely to hypothesis generation in this work.

### MLLMs in science

Many works consider multimodal VQA in scientific or medical domains like microscopy images [50], biological images [57], medical images [30, 34], and chemical structure drawings [43]. Still, they mostly test more straightforward perception and recognition. Figure comprehension for scientific articles does require more reasoning with multimodal data [41, 45–47, 61]; however, the images here are curated for publication, and the emphasis is on understanding content, rather than performing practical tasks. On the other hand, our benchmark includes realistic experiment images and tests more advanced reasoning like hypothesis generation and experiment planning. Scientific tasks have received much more attention in language-only LLMs [9, 15, 22, 37, 41, 58, 62, 63, 66, 67, 73, 80, 95], with GPQA being a notable work targeting PhD-level multi-step reasoning [62], but these do not test multimodal reasoning, which is important to many scientific areas [26]. In terms of models, most MLLMs in the biomedical domain are targeted at clinical tasks [34, 42, 59, 65, 75, 92].

### Multiple-choice question construction

A challenge in creating exams for education research and benchmarks in machine learning is generating multiple-choice questions with quality distractors [4, 27]. Recent works have explored LLM-based generation methods, including zero-shot distractor prompting, few-shot prompting, LLM fine-tuning with high-quality examples, and prompting for known error types [10, 23, 24, 94]. However, a major challenge is that generated distractors are not sufficiently vision-centric – MLLMs can often select the correct answer from the distractors without access to the image [74]. While this is partly explained by language biases on truly vision-centric MCQs [7, 29], others likely do fail to test visual understanding due to poor MCQ construction [41] – this informs our RefineBot MCQ generation method in Sec. 4.2.

## 3. The MicroVQA benchmark

Here, we present the MicroVQA benchmark for visual-question-answering (VQA) which advances multimodal scientific reasoning by tackling two major evaluation challenges. First, defining tasks is challenging because scientific research encompasses many complex activities [33] – we therefore define three important tasks important to biomedical discovery. Second, VQA samples cannot be readily sourced from existing exams or classification datasets – we therefore assembled a team of biologists to curate challenging reasoning problems. In Sec. 3.1, we provide an overview of the MicroVQA benchmark; Sec. 3.2 details the key tasks; and finally, Sec. 3 demonstrates that MicroVQA is at the forefront of measuring research-level reasoning in MLLMs. In the later Sec. 4, we describe our approach for generating multiple-choice questions.

### 3.1. Overview of MicroVQA

MicroVQA is a comprehensive dataset of 1,042 VQA triplets, manually curated by a team of expert biological researchers from diverse fields, and key attributes are summarized in Tab. 1. These expert-crafted questions test reasoning across three key tasks: expert understanding, hypothesis generation, and experiment proposal, and we additionally provide a taxonomy of sub-tasks in Sec. 3. The questions cover a broad spectrum, ranging from biological issues, such as “What is unusual about the cell shape?” – to technical imaging challenges like “Is this feature due to low signal-to-noise ratio (SNR) or is it real?”. We provide the ‘raw’ questions and answers written by experts, which are often long and nuanced, along with converted multiple-choice questions (MCQs) suitable for MLLM evaluation.

The images encompass the most common microscopy modalities used in human biology: brightfield, fluorescence, and electron microscopy. The sample types span the full range of microscopic scales – tissue, cellular, subcellular, and atomic – and emphasize organisms relevant to human biology and medically motivated tasks, namely human and mouse. Over 60% of the samples have multiple images because comparison is essential in microscopy research, and because multichannel fluorescence images cannot be represented in standard RGB. All these attributes are provided as metadata tags to facilitate deeper error analysis, thereby enhancing MicroVQA’s value as a resource for advancing AI capabilities in scientific research.

### 3.2. MicroVQA scientific reasoning tasks

To define specific reasoning tasks aimed at advancing biomedical research, we established the following criteria: (1) the tasks should use image-text inputs and text outputs suitable for MLLMs, (2) they should require higher-order reasoning beyond simple image processing, and (3) they should emphasize core experimental activities such as experimentation, analysis, and action [33], rather than auxiliary tasks like literature review or writing. The tasks were developed through interviews with nine co-author PIs and postdocs specializing in microscopy research (Appendix D). We identified three essential capabilities that we now introduce, with examples shown in Sec. 3.

#### Expert visual understanding

After performing an experiment, scientists must interpret data within its experimental context. In biological microscopy, this involves identifying patterns like protein distributions or cell morphologies, while also assessing technical aspects such as artifacts and noise levels. The task requires MLLMs to perform anomaly detection and image comparison, going beyond simple perception in two key ways: analysis must consider sample preparation context, and expert knowledge is needed to evaluate biological features and technical artifacts.

#### Hypothesis generation

The next step is proposing mechanistic hypotheses to explain experimental data. For example, when a drug treatment causes mitochondrial fragmentation in cells, a hypothesis might be that the drug disrupted calcium homeostasis, activating the DRP1 protein through a multi-step pathway, leading to fragmentation. This requires *abductive reasoning* [8, 20, 32, 81] as one must select from many possible hypotheses given incomplete information. The process demands the MLLM to integrate relevant domain knowledge with experimental context and visual features to reason about which hypothesis best explains the observations.

#### Experiment proposal

The final step is designing experiments to validate hypotheses. In microscopy, this often involves selecting appropriate assays and controls, requiring knowledge of suitable protocols and *deductive reasoning* about whether proposed experiments will provide evidence for or against the hypothesis. This task also may involve adjusting a particular experiment to address technical issues, like how to ensure a good signal-to-noise ratio, and this requires both image understanding and knowledge of protocols.

In Appendix F.7.1, we show an example question in each task with an expert-annotated ‘reasoning trace’ – it shows the type of reasoning required for typical questions.

### 3.3. Analysis of MicroVQA benchmark

Scientific discovery requires complex reasoning beyond basic perception and knowledge, and it requires expertise beyond school-level education. Here we show that MicroVQA addresses a gap that prior multimodal scientific benchmarks do not test *high level reasoning* at *research-level* difficulty.

**Figure 3.**
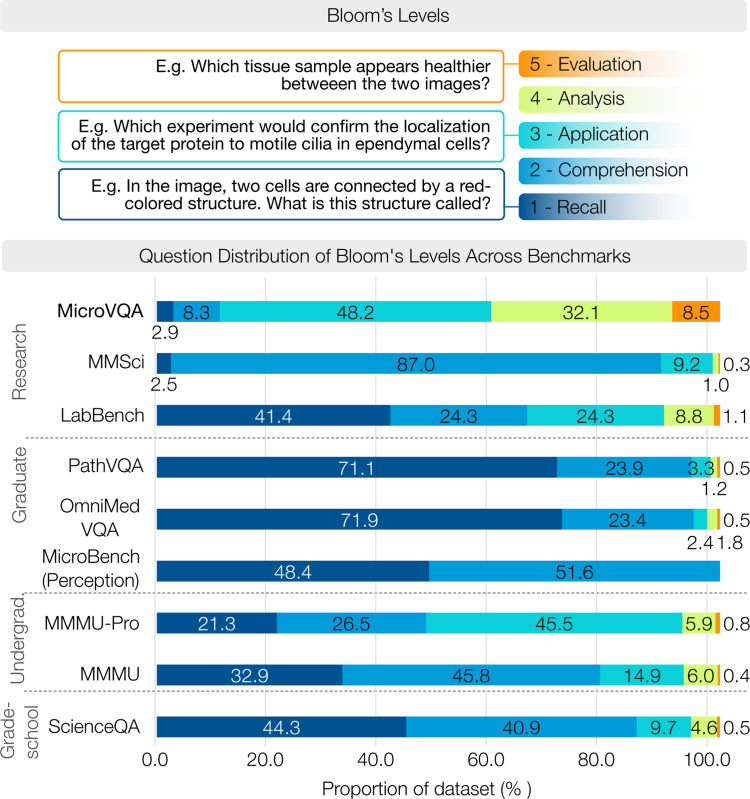
Composition of scientific MLLM benchmarks regarding estimated Bloom’s taxonomy [11]. Higher levels are more cognitively challenging. MicroVQA has more questions at higher levels compared to other benchmarks, for example, MMMU [87] and ScienceQA [53], while perception-driven medical benchmarks like OmniMedVQA are at lower levels.

To assess reasoning levels in MLLM benchmarks, we use Bloom’s taxonomy [11], which hierarchically classifies cognitive difficulty from recall to evaluation. We fine-tune GPT-4o to classify Bloom’s taxonomy on related MLLM benchmarks with results in Fig. 3; in Tab. 2 we additionally show key attributes of the same benchmarks. Fig. 3 shows that benchmarks having research-level and graduate-level difficulty are dominated by lower reasoning questions (levels 1–2), while our MicroVQA has higher level reasoning (levels 3–4). This is not surprising since OmnimedVQA and MicroBench derive questions from prior classification datasets, while our task definition and data collection approach does reflect high-level reasoning.

On the other hand, some benchmarks have higher Bloom’s reasoning level closer to MicroVQA – MMMU, MMMU-Pro, and Science-QA – but their difficulty level is lower – undergraduate or grade-school. The most comparable benchmark for reasoning is the undergraduate-level MMMU-Pro, and its dataset size is 1,730 compared to our 1,042: this shows that for reasoning-intensive benchmarks, sizes beyond 1,000 are very high.

## 4. MCQ generation process

### 4.1. Creation of raw VQA samples by experts

#### Expert generation of raw VQA samples

Creating VQA triples suitable for research-level reasoning tasks is time-consuming and requires expertise. We recruited 12 human experts to each create approximately 90 VQA samples, taking about 30–40 minutes per sample. Each submission included an image set, a question, a detailed response, and contextual information like experimental context and motivation. They are raw VQA triples, $\left(v_{0},q_{0},a_{0}\right)$ – ‘raw’ means the question and answer can be much longer and more detailed than typical VQA benchmarks. This allowed expert annotators to include enough detail to match what is a valuable input and response for an MLLM. Appendix E shows materials that prompt question creators. The images were sourced from the contributor’s own experiments, image databases, or research papers published since 2024, and all image licenses permit redistribution.

#### Quality control

We had three quality control criteria: questions should be challenging and not solvable with undergraduate-level knowledge; they should be aligned with the defined tasks; and there should be diversity in image content. Questions were reviewed against these criteria for each contributor, and feedback was provided for further VQA samples. These review rounds occurred after submitting the first 10 questions, and the first 40 questions.

### 4.2. MCQ generation from raw VQA samples

To benchmark MLLMs, we transform raw expert-created VQA samples to multiple-choice questions (MCQs) and propose new methods for MCQ generation.

#### Motivation: naive MCQ generation is inadequate

**Figure 4.**
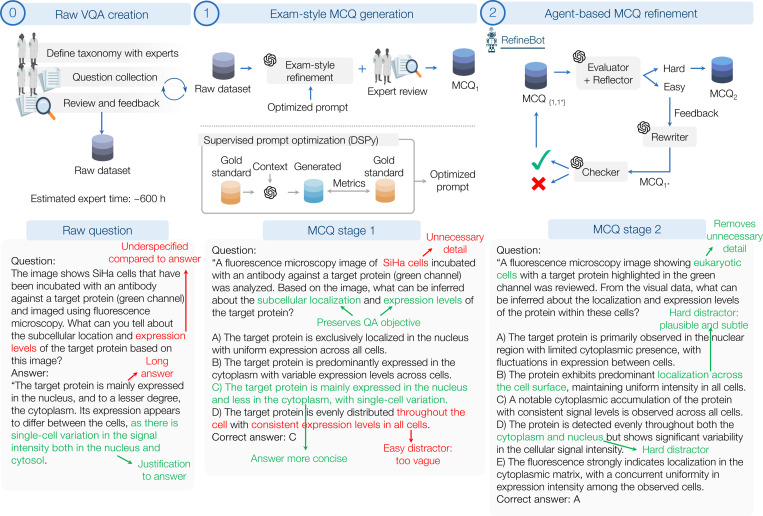
Constructing the MicroVQA multiple choice questions. (0) We defined tasks with domain biological scientists and created 1,061 raw VQA samples. (1) The raw samples were aligned to an exam-style MCQ by manually transforming a small set and optimizing an LLM prompt to match that alignment. (2) MCQs are further improved using RefineBot, a new iterative method to make MCQs more challenging. The lower panel shows an example MCQ from raw VQA to final: the annotations highlight key changes that we further explore in Appendix E.2, where red indicates issues, and green indicates good attributes.

The standard approach for VQA benchmarks is to zero-shot prompt an LLM with raw VQA samples [4], but this has two problems. The first is that generated MCQs do not follow established educational MCQ design principles (Appendix E.2), likely because the raw VQA questions and answers are often long and variable. Our stage 1 ‘exam alignment’, which we describe in the next section, addresses this challenge. The second challenge is that generated MCQs do not properly test MLLM capabilities. Evaluating GPT-4o on these MCQs scores 93% even *without the image*, despite most of the dataset questions clearly requiring image analysis, and we investigate this by inspecting the chain-of-thought outputs in Fig. 4 and Appendix E.2. Many distractors can be easily eliminated based on general biology knowledge, or because they are too vague compared to the correct option. We hypothesize that MCQ and distractor generation is challenging when the target answer string is long, and where the subject matter is specialized, suggesting that other scientific benchmarks may encounter similar issues. Overall, this motivates a more involved approach to distractor generation.

#### Stage 1: Exam-style MCQ generation

Here we ensured that multiple-choice questions (MCQs) conformed to established design principles for biomedical exams (Fig. 4). This effort was led by a physician-scientist co-author trained in medical board exam item writing, who reviewed educational literature on MCQ design [64] and Bloom’s Taxonomy for assessing cognitive skills in biomedical exams [5, 19, 89]. We began by manually transforming 50 user-submitted raw question-answer pairs, $\left(q_{0},a_{0}\right)$, into one-best-answer MCQs with k distractors, $\left(q_{1},a_{1},{\text{d}}_{1}\right)$. These ‘Gold Standard’ MCQs retained the original questions’ meanings while strictly adhering to NBME standards [64] and minimizing cues that enable test-wise guessing. We used these gold-standard MCQs in a supervised learning setup to develop a general LLM prompt, p, that maps raw question-answer pairs to exam-style MCQs. We leverage the DSPy framework [39, 40], which optimizes p to match the gold-standard MCQ outputs. Additionally, the optimization process incorporated supervision from LLM-based quality metrics: content similarity, NBME-aligned formatting, and the absence of extraneous clues. Further details are in Appendix E.2.

#### Stage 2: RefineBot question refinement

Although Stage 1 produces exam-aligned MCQs, many remain easily solvable due to language shortcuts (see Appendix D). To enhance difficulty, we introduce *RefineBot*, a method that increases MCQ complexity (Fig. 4). The key idea is that weaknesses in MCQ construction are revealed by the chain-of-thought (CoT) answers. The first LLM agent, the *evaluator/reflector*, answers the MCQ with CoT and then reflects on the strategies used. For example, a question about processes inside a virus might have a distractor referring to surface processes – this can be eliminated without referencing the image. The LLM summarizes its solution strategy into a reflection text: in this case, that the distractor was implausible based on knowledge that the stated process does not not occur inside the virus, and so does not match the question. It passes the reflection to the *rewriter* LLM agent. The rewriter revises the question stem and generates new distractors to invalidate the identified strategies: in this example it may create distractors referring to processes that do occur inside a virus. To prevent significant changes to the question-answer pair over iterations, we employ an LLM *checker* to ensure semantic consistency with the original pair. The revised MCQ is returned to the evaluator; if it still answers correctly, the loop continues. The process stops if the rewriter fails the *checker* or after n iterations. If it fails, RefineBot can be rerun with a different seed, often succeeding—a form of inference-time scaling [13]. All agents are GPT-4o-0806, but to mitigate potential bias against 4o in final evaluation, we also use Claude-3.5-Sonnet-0620 as the *evaluator* agent; we assess biases in the experiments section.

#### Final MCQ quality check

Since MCQ generation involves LLM processing, we need to verify the correctness of the final answer. Each MCQ is manually reviewed by the same expert who created that particular question (between 80 and 120 per person). For any question with issues, that expert makes minimal changes to the question correct it.

## 5. Experiments

### 5.1. Benchmarking MLLMs with MicroVQA

We evaluate state-of-the art multimodal large language models (MLLMs) on the MicroVQA benchmark. We include open and closed models spanning the categories: reasoning, large, small, and medical – medical is the closest domain to microscopy with specialist MLLMs. We utilize standard chain-of-thought prompting [88] (deatails in Appendix F). We report mean accuracy in multiple-choice VQA for each tasks Tab. 3. We also perform and analyze no-image Appendix F.5.

#### MicroVQA is challenging for all MLLMs

Our evaluation reveals a substantial gap between current MLLMs and the upper bound, with the leading model, o1, at 52.8. There is surprisingly little variation in performance between models, with most closed and open models all scoring above 40, with the lowest score (Llama-3.2–11b) likely due to instruction-following issues. A human baseline (Appendix F.3) scores only 50.3 – this shows that biology experts are specialized to subdomains, and are challenged by different subdomains. In fact this demonstrates that experts could benefit from MLLMs that can solve MicroVQA’s tasks. The performance variation across the 3 tasks is small – the highest score is 56.4 on ‘expert visual understanding (V)’, compared to the highest overall of 52.8. The ‘hypothesis generation’ task is the hardest for all models and the gap is strongest for smaller models. For example the gap between hypothesis generation and expert visual understanding is 3.7 for the strongest large model, but 10.7 on the strongest small model.

#### Smaller models are surprisingly competitive

For all models with a lower-parameter equivalent – Gemini-1.5, QwenVL, VILA, and Llama – the drop in performance due to size is less than 3 for all except Llama. Moreover, these drops are smaller than on other multimodal reasoning benchmarks like MMMU, where for example, Gemini drops by 9.6 between Pro and Flash-8B [87]. Typically, smaller models have the same vision encoder, but a smaller LLM [79]. Since the size of the language model has a small impact, we hypothesize that for the ‘solved’ part of MicroVQA, the language-only reasoning is relatively simple compared to domains like math that require multi-step logic. This suggests that other aspects of MLLM reasoning are more challenging, like multimodal reasoning. If true, this suggests that future work could focus on stronger image representations, and this idea is supported by the qualitative error analysis in Sec. 5.2.

#### Specialist biomedical training does improve performance

While no specialist MLLMs have been developed for microscopy research in particular, LLaVA-Med is fine-tuned on scientific articles from PubMed that include data types overlapping with MicroVQA – especially (tissue) pathology. In Tab. 3, we compare LLaVA-Med against it’s base model, LLaVA-Mistral-7B and find overall stronger performance by 4.5 points. This suggests two clear opportunities for improving MicroVQA performance. The first is to instruct-tune with a dataset that better aligns with the MicroVQA data domain: not only tissue pathology, but also fluorescence and electron microscopy, which is available in scientific articles [93]. The second is to simply begin training from a stronger open model like Pixtral. Beyond that, there is more to explore in MLLM specialization, such as tuning the base image encoder for microscopy [51].

#### RefineBot MCQ generation is very effective but introduces small model biases

We introduced a new method for refining MCQs to make them more challenging – RefineBot. Tab. 4 shows that between stage 1 and stage 2 (before and after RefineBot), the relative accuracy for all models drops by between 35% and 42%. Firstly, this validates that RefineBot is a valuable tool for making benchmarks harder. However, the most significant drops are for models used by RefineBot (GPT-4o & Claude-3.5-Sonnet, and their smaller versions, GPT-4o-mini & Claude-3-Haiku). The adversarial process introduced a small bias against these models (and we hypothesise the bias would be higher if using only one model instead of two). Despite making fair evaluation slightly more challenging, our use of frontier LLMs in the refinement process is well-motivated, as these models are best positioned to generate challenging, high-quality MCQs that probe the boundaries of current capabilities. Moreover, the bias seems modest – less than 10 points of relative drop – while the benefit is a huge increase in difficulty across all test MLLMs, as seen in Tab. 4.

#### Metadata-based analysis supports deeper error analysis

**Figure 5.**
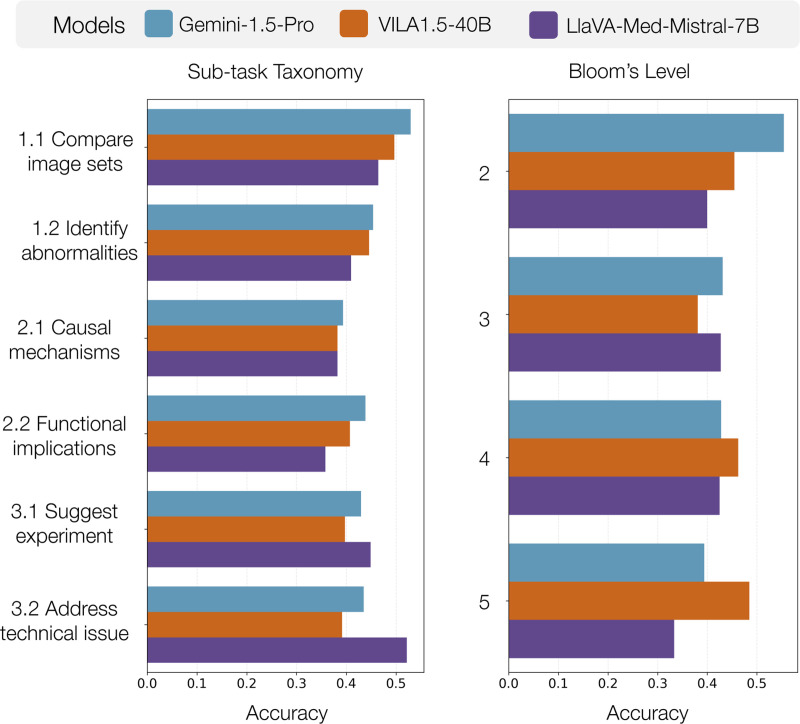
Performance by sub-task and Bloom’s level for best models: Gemini-1.5-Pro (closed source), VILA1.5–40B (open-source), and LlaVA-Med-Mistral-7B (medical).

Fig. 5 shows error rates based on attribute tag. In Appendix F we explore results further – namely that multi-image reasoning has surprisingly good performance, higher level Bloom’s questions are harder, and (not shown) rare image modalities perform worse.

### 5.2. Error analysis on MicroVQA

To understand model errors, three biomedical experts reviewed the Chain-of-Thought (CoT) responses of 30 samples on Claude 3.5 Sonnet, with each review requiring at least 45 mins of careful analysis. Errors were classified into a major category and minor contributing errors were also noted. Expert perception accounted for 50% of errors, 30% were knowledge errors, and 13% were due to overgeneralization; the final 7% were text hallucination or general reasoning error. Appendix F.6 shows examples for the most common types. We expand on this analysis with an automated version across the complete dataset in Appendix F.7.

**Expert perception errors** occurred when the model misinterpreted visual features, leading to incorrect inferences. For example, in Fig. 13, the model judges the small electron-dense objects as ribosomes, rather than stress granules – ribosomes tend to be smaller, have a more regular shape, and lower contrast. Language bias may have contributed to the preference for ribosomes since they are very commonly studied in EM literature available to LLM training. Overall, perception errors were the dominant error type (50%), which suggests that future methods should improve vision representations in MLLMs for microscopy.

**Knowledge errors** highlight gaps in nuanced biomedical knowledge. One example in Fig. 17 deals with understanding how protein localization changes during cell signaling. An experiment gives information about a transmembrane signaling protein, and the question asks to interpret the image and explain the function in these cells. The model correctly perceives a punctate distribution, but assumes the role of coordinating intracellular vesicle trafficking. This represents a shallow interpretation that could have been improved with specialized biomedical knowledge. These errors could be mitigated by improving knowledge in MLLMs, either in training or as a database at inference.

**Overgeneralization errors** (and simplification errors) reflect the model’s tendency to apply broad scientific principles without regard for specific context – arguably they are *reasoning* errors. In one example in Fig. 21, the question is to compare the risk of malignancy of a tumor, schwannoma, to other nerve sheath tumors. During reasoning, the model proposes a simplified question – what is true about schwannoma – which it answers while ignoring the comparison.

## 6. Conclusion

MLLMs hold great potential for advancing scientific discovery. We aim to inspire the development of broadly applicable scientific AI systems. Future directions include training models for more robust image representations, integrating knowledge bases, exploring open evaluation methods, and studying LLMs’ reasoning over hypotheses. Beyond microscopy, we hope our benchmark serves as a blueprint for AI evaluation in expert-driven domains. More generally we aspire for different application domains – biomedicine, chemistry, materials science – to share methods towards the common goal of building scientific AI systems.

## Supplementary Material

Supplement 1

## Figures and Tables

**Figure 1.**
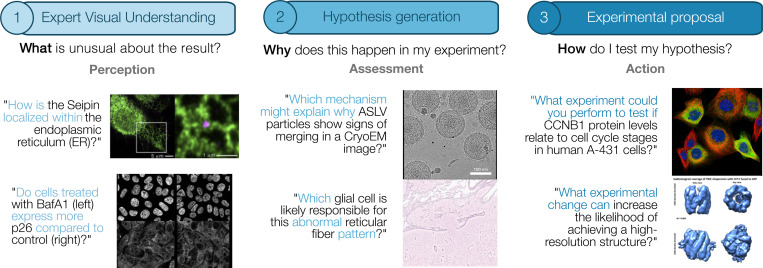
A scientific experimentation workflow drives discovery: researchers analyze experiments, develop hypotheses, and design further experiments to test their ideas. We release MicroVQA, a visual question answering (VQA) benchmark to test these three tasks in the context of biological microscopy. Each of the 1,042 samples is created by a biology expert, and transformed into a multiple choice question (MCQ).

**Figure 2.**
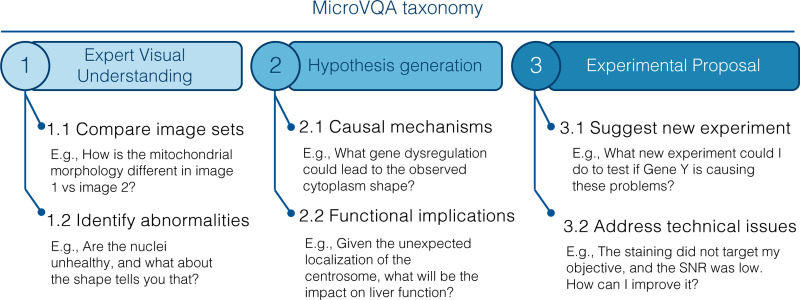
MicroVQA taxonomy of sub-tasks.

**Table 1.** MicroVQA benchmark attributes.

Dataset feature	Value
Total questions	1,042
Multi-image questions	423

Avg. MCQ question length	66
Avg. MCQ answer length	15
Avg. raw question length	158
Avg. raw answer length	52

Unique image sets	255
Image Modalities	Light, Fluoro, Electron
Image Scales	Tissue, Cell, Subcell, Atomic

Organisms	31
Research areas	33

Expert question creators	12
Time to create 1 question	30–40 mins
Time to quality check 1 MCQ	5 mins

**Table 2.** Comparing scientific multimodal benchmarks close to MicroVQA for in terms of reasoning level or difficulty beyond college level. We show difficulty level, scientific domain, dataset source, and size. Compared to others, MicroVQA either has higher difficulty level, or it has higher reasoning level (which is established in Fig. 3). Compared to others at the same reasoning level, namely MMMU-Pro, it has similar size.

Benchmark	Difficulty level	Domain	Source	Size
**MicroVQA**	research	microscopy	expert-curated	1,042
MMSci [47]	research	science	paper figures	7132
LabBench [41]	research	biology	webQA	181
PathVQA [30]	graduate	pathology	texbooks	16.3k
OmnimedVQA* [34]	graduate	medical	classification dataset	127.9k
Microbench [50]	graduate	microscopy	classification dataset	17.2k
MMMU [87]	undergraduate	general	textbooks, webQA	11k
MMMU Pro [88]	undergraduate	general	MCQ dataset	1,730
Science QA [53]	grade-school	science	exams	16.8k

**Table 3.** VQA accuracy on MicroVQA by task: expert visual understanding (V), hypothesis generation (H), experiment proposal (E). Models marked * were used in MCQ generation, which may affect comparative performance (see Sec. 5.1). The model ^†^ is the base LlaVA for LLaVA-Med. Best values are bolded.

	Model	Overall	V	H	E
R	o1 [36]	**52.8**	55.4	**50.2**	**53.0**

large models	*Claude-3.5-Sonnet [6]	51.7	54.1	**50.2**	50.4
	Gemini-Pro-1.5 [69]	51.1	52.0	**50.2**	50.9
	Pixtral-Large [2]	49.8	50.8	49.5	48.7
	Grok-2-Vision [84]	48.4	50.3	46.4	48.7
	Qwen-2-vl-72b-Instruct [79]	47.5	49.2	45.7	47.8
	VILA1.5–40b [48]	47.5	47.2	47.9	47.4
	*GPT-4o [1]	45.6	48.7	43.1	44.8
	Llama-3.1-Nemotron-70b-Instruct [83]	44.2	44.9	43.3	44.8
	Llama-3.2–90b-Vision-Instruct [21]	42.4	44.9	42.1	38.7

small models	Qwen-2-VL-7b [79]	48.8	54.1	43.3	49.6
	Claude-3.5-Haiku [6]	47.1	48.0	43.8	51.7
	Gemini-Flash-1.5–8b [69]	46.7	48.7	43.6	49.1
	GPT-4o-mini [1]	46.2	48.5	43.6	47.0
	Pixtral-12b [2]	45.6	46.9	44.8	44.8
	VILA1.5–13b [48]	41.8	41.8	47.5	40.9
	Llama-3.2–11b-vision-instruct [21]	30.3	32.4	29.3	28.7

LLaVA-Med-Mistral-7B [42]	43.0	37.3	47.1	41.6
medical	LLaVA-Mistral-7B [49]	39.8	31.6	43.1	37.1

	Random	22.0	21.9	21.8	21.9
	Human	50.3	52.7	47.5	51.4

**Table 4.** Ablation study on MicroVQA MCQ generation stages (shown in Fig. 4). Accuracy is high because MCQs have shortcuts (Sec. 4.1) after ‘Stage 1’ exam alignment, but is lower after ‘Stage 2’. Final column is the relative decrease in accuracy. Models with * were used in Stage 2 generation and have the biggest accuracy drops (**bolded**). They are grouped with different models from the same provider in **.

	Stage 1	Stage 2	Relative drop
*GPT-4o	79.7	46.8	−41.2
**GPT-4o-mini	75.6	46.2	−39.0
*Claude-3.5-Sonnet	82.2	51.7	−37.1
**Claude-3.5-Haiku	77.3	47.1	−39.0

o1	81.6	52.8	−35.3
Pixtral-Large	80.1	49.8	−37.8
Gemini-Pro-1.5	79.1	51.1	−35.4
