# Supplementary material for: MicroVQA: A Multimodal Reasoning Benchmark for Microscopy-Based Scientific Research
Source: ArXiv. 2025 Mar 17:arXiv:2503.13399v1. Preprint. [Version 1] (PMC11957224)
Supplement: Supplement 1 [file NIHPP2503.13399v1-supplement-1.pdf]

MicroVQA: A Multimodal Reasoning Benchmark for Microscopy-Based Scientific Research

Supplementary Material

Appendices A-C include acknowledgments, author contributions, ethics, and discussion of limitations and future work. The remaining sections correspond to sections in the paper. Appendix D is benchmark details from the main Sec. 3. Appendix E is MCQ generation details from the main Sec. 4. Appendix F is the experiments details from the main Sec. 5.

Table of Contents

A. Acknowledgments and author contributions **1**

B. Ethics **2**

C. Limitations and future work **2**

D. Benchmark details **3**
- D.1. Accessing MicroVQA benchmark . . . 3
- D.2. Dataset Schema and Structure . . . 3
- D.3. Training contamination mitigation . . . 3
- D.4. Benchmark attribute details . . . 4
- D.5. Related benchmarks . . . 4
- D.6. Bloom's taxonomy . . . 5
- D.7. Methodology for proposing tasks . . . 6
- D.8. Guidance for question creators . . . 7
- D.9. Microscopy image representations . . . 7

E. MCQ generation details **7**
- E.1. Question collection process details . . . 7
- E.2. Experiment on naive MCQ generation . . . 7
- E.3. Stage 1 MCQ generation (exam alignment) . . . 8
- E.4. Stage 2 MCQ generation (RefineBot) details . . . 8
- E.5. MCQ generation results: stage 1 exam alignment . . . 13
- E.6. MCQ generation results: stage 2 RefineBot . . . 13

F. Experiments details **15**
- F.1. Evaluation prompts . . . 15
- F.2. Model details . . . 16
- F.3. Human baseline on MicroVQA . . . 17
- F.4. More experiments on attribute-level information . . . 17
- F.5. Language shortcut ablations . . . 17
- F.6. Error analysis . . . 20
- F.7. Reasoning trace analysis . . . 31

A. Acknowledgments and author contributions

Acknowledgements We thank Christian Kindermann for feedback on motivation for benchmarking and reasoning taxonomies; Siddharth Doshi for feedback on motivation for applications in scientific chat systems; and Krisjanis Mazurs for feedback on structuring user interviews. We also thank Assaf Zaritsky, Andrew S Moore, Pedro Guedes Dias, Angus Toland, Helen Healy, and Andrew Kassianos, and Loic Royer for discussions on defining the VQA tasks.

Funding acknowledgements We gratefully acknowledge funding from HAI Hoffman-Yee (E.L., S.Y-L., J.M.H.B.), Schmidt Futures (E.L.), the Bridge2AI Program (NIH Common Fund; OT2 OD032742; E.L.,), Göran Gustafsson Foundation and Knut and Alice Wallenberg Foundation (KAW 2021.0346; E.L.) as well as to MU (HPA). S.C. received funding from NIH/NIGMS R35GM133460 and CZI A23-0264-001. J.N.H. was supported by a Postdoctoral Fellowship from EMBO Postdoctoral Fellowship (ALTF 556-2022).

Author contributions

- Project conception: JB, JN, LBS, AL, SYL
- Defining the VQA tasks: JB, JN, JGG, WC, SC, JNH, CL, SYL
- Managing benchmark collection processes: JN, JB
- Creating benchmark questions: DB, ZC, SMH, AJ, WDL, MGN, RY, CZ, JNH, CL
- MCQ conversion pipeline: JB, JN, LBS
- Evaluations: JB, SRG, YS
- Qualitative error analysis: JN, JGG, CZ
- Quantitative error analysis: LBS
- Paper writing and figures: JB, JN, LBS
- Supervision: SYL, EL, CL, MDL, CNH, SC, WC

B. Ethics

Ethical use of biomedical data: MicroVQA was developed with a commitment to ethical practices in handling biomedical research data. All microscopy images included in the dataset were either original, unpublished data or sourced from open-access articles (e.g., CC-BY licensed) published after January 2024. The dataset does not include patient-identifiable information, and MicroVQA adheres to applicable privacy and research ethics guidelines. The dataset does not constitute human subjects research.

Potential societal impacts: Biomedical AI models, including those trained on MicroVQA, have the potential to influence research and healthcare significantly, both positively and negatively. While these models can accelerate scientific discovery, they may also perpetuate biases in training data or result in uneven performance across demographic or biological groups. To mitigate these risks, MicroVQA includes metadata annotations to support the analysis of potential biases and emphasizes diverse data sourcing. We will engage with the research community to address emerging ethical concerns.

Data licensing and usage: MicroVQA is distributed under the Creative Commons Attribution-ShareAlike 4.0 International (https://creativecommons.org/licenses/by-sa/4.0/) license to promote transparency and collaboration in the research community. This license reflects our efforts to balance open access with the ethical use of data, enabling both academic and commercial applications of the dataset while adhering to original license requirements. Full licensing details are available at https://creativecommons.org/licenses/by-sa/4.0/.

C. Limitations and future work

While MicroVQA advances research-level reasoning benchmark significantly, we can identify a number of limitations and avenues for future work.

Open vs closed evaluation Like most VQA benchmarks, we used multiple-choice (MCQ) evaluation. However downstream applications will obviously operate in an open setting, without options and requiring more detail. MLLM research does have some open benchmarks [86], though impartial evaluation is a significant challenge. Our early experiments with open prompting suggest that models like GPT-4o tend to give very vague answers, and tend to depend strongly on the text part of the input prompt.

Dataset scale Our final dataset had 1042 samples, which is smaller than some other VQA benchmarks like MicroBench [50] and OmniMedVQA [34]. MicroVQA's scale sufficient to compare overall scores between models, however statistical power starts to become limiting when trying to compare different models on subsets of the dataset. How does this compare to other benchmarks? As we can see from Tab. 2 and Fig. 3, the larger datasets tend to be those with lower-level reasoning according to Bloom's taxonomy. Their samples tend to be derived automatically from metadata tags or classification labels. Benchmarks with comparably high-level reasoning, like MMMU-Pro, have a similar scale to ours (1,700). In the language-only domain, the research-level reasoning benchmark GPQA has 448 samples. This trend reflects a clear, practical limitation: it is very time-consuming for humans to create difficult reasoning questions, and the pool of capable experts is small.

Breadth of dataset attributes in microscopy We aimed for MicroVQA to cover a broad range of the microscopy discipline; for example, we have samples from all four relevant scales – tissues, cells, subcellular, atomic. However, each human annotator is an expert in a subset of microscopy and biology, so there are practical limits on dataset coverage. We, therefore, chose to focus on the most common imaging modalities – brightfield, fluorescence, electron – while skipping less common modalities like Raman spectroscopy. In terms of sample types, we emphasized those relevant to humans – human and mouse – while having only a few samples from rare organisms like bottlenose dolphin.

Coverage of scientific reasoning Looking even more broadly, the motivation of this work is to advance reasoning in scientific research generally. However, we use microscopy in particular. This is because of a clear trade-off in breadth vs depth. Our dataset strikes a good balance, given that microscopy represents a large portion of visual data in biomedicine and biology. Future work can reuse our framework to generate reasoning VQA benchmarks in other fields of visual biomedicine and biology, such as in medicine [34] or ecology [93].

Resolving MCQ shortcuts A key contribution in this paper is the RefineBot for generating multiple choice questions (MCQs) that are too challenging to solve using text-only shortcuts. Our approach used the outputs of existing MLLMs as signal to make distractors harder. However, this cannot guarantee the removal of shortcuts; more generally, harder distractors may exist. Therefore, the next generation of LLMs may be able to identify and exploit new shortcuts. If that occurs, we can run RefineBot again using the new LLM, creating MicroVQA-2. This strategy could be used for other VQA evaluations.

D. Benchmark details

D.1. Accessing MicroVQA benchmark

MicroVQA is an expert-curated benchmark for multimodal scientific reasoning, specifically in microscopy-based biological research. The dataset comprises 1,042 multiple-choice questions (MCQs) created by biological researchers to represent authentic scientific practice. Each MCQ is paired with an associated microscopy image and metadata describing the experimental context. A demonstration subset of 28 instances is available for initial exploration.

The MicroVQA dataset is publicly accessible on HuggingFace at https://huggingface.co/datasets/jmhb/microvqa. It is distributed under the Creative Commons Attribution-ShareAlike 4.0 International (CC BY-SA 4.0) license, allowing reuse and share-alike redistribution with attribution. The dataset is available in Apache Arrow and Parquet formats for efficient data handling. These formats support fast serialization, memory mapping, and streaming from the web, facilitating seamless integration into machine learning pipelines.

D.2. Dataset Schema and Structure

The dataset schema defines the fields included for each data instance. Figure 6 provides a schema summary, while a sample data instance is detailed below 7.

```
{
    "image_id": datasets.Value("string"),
    "image": datasets.Image(decode=True, id=None),
    "label": datasets.ClassLabel(),
    "label_name": datasets.Value("string"),
    "domain": datasets.Value("string"),
    "subdomain": datasets.Value("string"),
    "modality": datasets.Value("string"),
    "submodality": datasets.Value("string"),
    "stain": datasets.Value("string"),
    "microns_per_pixel": datasets.Value("string"),
    "questions": datasets.Value("string"),
}
```

Figure 6. Example data schema.

D.3. Training contamination mitigation

To mitigate contamination, all data in MicroVQA were sourced from original, unpublished microscopy images or open-access articles (e.g., CC-BY-licensed) published after January 2024. Additionally, canary strings were embedded in the dataset to detect potential contamination during fine-tuning or model evaluation.

```
{
    "image_id": "d54bb7ec-284f-4218-a47d-af87bb371de5",
    "image": datasets.Image(decode=True, id=None),
    "label": datasets.ClassLabel(),
    "label_name": "pathology",
    "domain": "pathology",
    "subdomain": "gastrointestinal pathology",
    "modality": "light microscopy",
    "submodality": "brightfield microscopy",
    "stain": "H&E",
    "microns_per_pixel": "Not provided",
    "questions": "Question:
        ```A hematoxylin and eosin (H&E)-stained micrograph shows cellular
        formations within a gastrointestinal tissue sample with unique
        organization and cellular patterns. What pattern and morphology do these
        cells most closely align with?

           A) Bundles of elongated cells with pale pink cytoplasm and uniform oval
              nuclei in a crisscross pattern
           B) Circular groups of cells with mucin in central spaces and small,
              dark nuclei
           C) Dense arrays of polygonal cells with small nucleoli and vacuolated
              cytoplasm
           D) Rows of cells with conspicuous cytoplasm and rounded nuclei
           E) Diffused arrangement of cells with transparent cytoplasm
              and irregularly clustered chromatin

        Correct Answer: A) Bundles of elongated cells with pale pink cytoplasm
        and uniform oval nuclei in a crisscross pattern```
}
```


Figure 7. Example data instance.

D.4. Benchmark attribute details

The MicroVQA benchmark is designed to evaluate multimodal reasoning capabilities in biomedical research contexts. The dataset incorporates diverse attributes such as organism, research subject, imaging modalities, and biological length scales (image scales) to ensure a comprehensive assessment1. These metadata not only enhance the scientific relevance but also enable analysis of reasoning across different biological and microscopy contexts. This section describes the methodology used to define, compute, and annotate these key attributes.

The benchmark comprises 1,042 total questions, including 423 questions containing multiple images combined into panels. A collage of sample images is shown in 25. The average MCQ token lengths were computed using the `o200k_base` tokenizer, with separate calculations for the raw input and formatted multiple-choice questions. Imaging modalities are broadly categorized into light, fluorescence, and electron microscopy. Biological length scales span four levels: tissue (millimeter scale), cellular (micrometer scale), subcellular (nanometer scale), and atomic (angstrom scale), enabling reasoning across diverse structural hierarchies.

To annotate attributes such as organism, specimen, and research subject, we manually reviewed random samples from the dataset and created a taxonomy to cover the most likely categories. A biomedical domain expert manually labeled these categories for 50 raw input question-answer pairs and performed initial prompt engineering. We used DSPy to optimize the prompt and few-shot examples on the labeled dataset. The input context included the taxonomy of organisms8 and research subjects 9.

These annotations were the gold standard to guide DSPy prompt engineering for automated attribute labeling on the full dataset. Prompt instructions included fallback rules (e.g., tagging "None" for indeterminate attributes) to ensure accuracy and minimize hallucination. The optimized pipeline annotated all questions while ensuring the validity of the dataset attributes.

D.5. Related benchmarks

In section 3.3 and Tab. 2 we compare MicroVQA to related scientific multimodal benchmarks, focusing on its intersection of high-difficulty and advanced reasoning questions. Beyond these key attributes, we examine the sources of the questions. Most benchmarks rely on existing question repositories, such as exams, web-based QA datasets, textbooks, or other MCQ datasets. This approach bypasses the need for MCQ generation methods, as the questions and options are pre-tested and curated, making these benchmarks more representative of established tasks. Another common strategy involves reformatting classification datasets into MCQs using question templates, which increases task diversity but often limits scenario unique-
```

```
Taxonomy of organisms in biology| # Taxonomy of Organisms in Biology
and Biomedical Research:
Apis mellifera (Honeybee)
    Social insect with complex behavior.
    Keywords: honeybee, social behavior, ecology, pollination.

Arabidopsis thaliana (Thale Cress)
    Model plant organism.
    Keywords: Arabidopsis, plant genetics, photosynthesis.

Caenorhabditis elegans (C. elegans)
    Transparent nematode worm.
    Keywords: worm, development, aging, programmed cell death.

Danio rerio (Zebrafish)
    Fish with transparent embryos.
    Keywords: zebrafish, vertebrate development, embryology.
...

Drosophila melanogaster (Fruit Fly)
    Insect with rapid life cycle.
    Keywords: fruit fly, genetics, development.

Escherichia coli (E. coli)
    Bacterium used in genetics and molecular biology.
    Keywords: bacteria, gene expression, plasmids.

...
Homo sapiens (Human Cells)
    Cultured human cells (e.g., HeLa, HEK293, A431, U2OS).
    Keywords: human cell lines, immortalized cell lines

In vitro (None)
    Purified molecules and reactions in controlled environments.
    No living cells or organisms.
    Keywords: purified proteins, purified molecules, cell-free systems
...
```

Figure 8. Examples of taxonomy classes used as context to LLM to assign an organism to a question. A YAML file with the full taxonomy will be released with the code.

ness and language variability. In contrast, MicroVQA is built by directly querying experts for challenging, domain-specific research questions. By starting with long form questions and answers, our approach requires an innovative MCQ generation method, ensuring uniquely tailored questions that push the boundaries of domain-specific reasoning and knowledge assessment.

D.6. Bloom's taxonomy

Bloom's Taxonomy is a model for evaluating cognitive skill levels in educational assessments. It has six hierarchical levels of learning: recall, understand/comprehend, apply, analyze, evaluate, and create. Multiple-choice questions can test five of Bloom's levels but not the sixth level, "create". These levels provide a structured framework for assessing lower-order and higher-order thinking skills, which are essential for designing robust benchmarks like MicroVQA.

Initial attempts to use LLMs to classify Bloom's levels for biomedical visual question answering (VQA) benchmarks revealed a systematic overestimation of cognitive skill levels. This discrepancy limits the naive use LLMs for automated Bloom's classification, perhaps due to the nuances of applying Bloom's taxonomy in domain-specific contexts. To ensure accurate classification of Bloom's level, a physician-scientist with NBME training in item writing and familiarity with Bloom's classification manually labeled Bloom's level for 200 examples from public MCQ questions, MicroVQA, MicroBench, and ScienceQA. These examples were used to fine-tune GPT-4o-mini (gpt-4o-mini-2024-07-18) to classify Bloom's level and align with expert judgments.

The fine-tuned Bloom's classifier was applied to MicroVQA and numerous public science/biomedical benchmarks to assess the cognitive skills evaluated by each question. Each question was mapped to one of Bloom's levels, providing insights into the distribution of cognitive tasks across the benchmark. This analysis revealed that while existing benchmarks predominantly tested lower-order skills (e.g., recall and comprehension), MicroVQA has a greater emphasis on questions that require higher-order reasoning (e.g., analyzing, evaluating), filling a critical gap in multimodal scientific reasoning

```
Taxonomy of research subjects| #Taxonomy of Research Subjects in Biology:
Anatomy
    Study of the structure of organisms and their parts.
    Keywords: organs, tissues, morphology, physical organization.

Biochemistry
    Chemical processes within living organisms.
    Keywords: proteins, enzymes, nucleic acids, metabolic pathways.
...

Cell and Molecular Biology
    Structure and function of cells and their molecules.
    Keywords: cellular processes, gene expression, molecular pathways.

...
Developmental Biology
    Process by which organisms grow and develop.
    Keywords: embryology, morphogenesis, genetic regulation.
...
Genetics
    Study of heredity and gene function.
    Keywords: DNA, inheritance, genetic variation, gene expression.

Immunology
    Immune system in health and disease.
    Keywords: antibodies, immune response, pathogens, vaccines.

Microbiology
    Study of microorganisms.
    Keywords: bacteria, viruses, fungi, parasites, microbial ecology.

Neurobiology
    Structure and function of the nervous system.
    Keywords: neurons, brain, neural circuits, cognition.
...
Structural Biology
    Molecular structure of biological macromolecules.
    Keywords: protein folding, nucleic acid structures, crystallography.
...
```

Figure 9. Examples of taxonomy classes used as context to LLM to assign a research subject to a question. A YAML file with the full taxonomy will be released with the code.

assessments.

D.7. Methodology for proposing tasks

Here, we expand on the task proposal methodology introduced in Sec. 3.2. To define specific reasoning tasks aimed at advancing biomedical research, we conducted a comprehensive expert consultation process. Through structured interviews with nine Principal Investigators and postdocs specializing in microscopy research, including participants from both academia and industry, we developed a framework for tasks that would meaningfully integrate multimodal large language models (MLLMs) into microscopy research workflows.

The task selection process followed specific criteria: tasks must utilize image-text inputs and text outputs suitable for MLLMs; they should require higher-order reasoning beyond simple image processing; and they should emphasize core experimental activities (experimentation, analysis, and action) rather than auxiliary tasks like literature review or writing [33].

Our methodology involved a two-phase interview process. The first phase consisted of initial one-hour individual sessions with eight research groups featuring open-ended, unstructured discussions about key challenges in microscopy experiments, potential applications of MLLMs, current workflows and unmet needs, and possible AI system integration points. One week later, we then conducted follow-up interviews to consolidate findings and build consensus.

To avoid anchoring biases, we intentionally withheld predefined categories during initial interviews. This approach enabled unbiased task identification and allowed us to capture a broad spectrum of potential use cases. Through this iterative process, we identified three essential capabilities that align with the scientific life cycle for microscopy experiments: advanced image interpretation, hypothesis generation, and experimental design/hypothesis testing.

D.8. Guidance for question creators

Question creators were given detailed guidance as pdfs, that we share at https://github.com/jmhb0/microvqa under folder `benchmark/human_collection`. During the submission process, we completed two rounds of quality control to ensure difficulty and alignment to the prompts, as described in Sec. 4.1.

D.9. Microscopy image representations

MLLMs are trained primarily on three-channel RGB images, however microscopy images are more diverse. To ensure alignment with how common MLLMs process images, we required that each image artifact for a single question was represented as no more than six RGB images. Where multiple images were needed, they could be multiple image files, or a single image that is concatenated. For videos, users could submit up to six frames; for 3d, they could submit slices or z-projections.

An important difference is multichannel images. Many fluorescence microscopy images are multichannel, where the image collection ensures that each channel represents a certain structure. For example channel 0 may be nucleus, and channel 1 may be mitochondria. There are a few approaches. Some questions represent this situation as separate grayscale images for each channel. Others show a 'composite image', which is a single image where one each channel is mapped to one of the RGB channels; e.g. a common visualization will put nucleus in blue and mitochondria in green. Some questions do both: grayscale images for each channel, along with a composite image merging them.

Finally, we allowed screenshots from research papers or image software, since this is a realistic way that future LLM-users would use AI tools.

E. MCQ generation details

E.1. Question collection process details

Data collection used a structured submission form shared with collaborators. The form required users to provide the following:

- Image or Image Set: Users uploaded microscopy images, optionally including multiple images for comparative questions.
- Experiment Details: A brief description of the experimental setup, imaging modalities, and any relevant biological context.
- Question Text: Each question was aligned with one of three predefined use cases: identifying interesting features, explaining mechanisms, or proposing next steps.
- Answer and Distractors: Contributors provided a correct answer and biologically plausible incorrect answers.

Quality control was performed in multiple rounds. To familiarize users with the task and data requirements, each user submitted an initial form with 4-6 questions for manual quality control and feedback. Feedback was provided to users on improving question specificity, ensuring the questions required image interpretation rather than relying solely on general knowledge. Contributors were encouraged to refine their submissions based on the feedback to better align with the tasks and goals of the MicroVQA benchmark.

Users were encouraged to submit data from multiple sources, imaging modalities (e.g., fluorescence, electron microscopy), and biological scales (e.g., tissue, cellular, subcellular) to promote diversity. Contributors could upload microscopy images from their experiments or use CC-BY publicly available images. Common image sources included preprints or publications released after January 2024 (to mitigate contamination), or from established image repositories [14, 17, 18, 31, 72, 76]. This ensured that all images were either original or sourced from open-license publications, enabling their use in an open-source benchmark.

E.2. Experiment on naive MCQ generation

In Sec. 4.2, we discuss how the naive approach to MCQ generation – simply zero-shot prompting GPT – leads to MCQs that are very easy for frontier models to solve. Which ultimately motivated our two-stage MCQ generation approach. Here, we discuss those experiments in more detail. We create a naive version of MicroVQA using a standard prompt [34, 82]:

```
You are an expert in molecular and cell biology, and in microscopy.

I will give you an original biology-related question and its answer, your task is to rephrase an equivalent
question with identical answer. The question related to an image, and we don't show the image.
Meanwhile, I want to transfer this QA-pair into a multi-choice question. Please generate 5 incorrect options to
construct the candidate options.

{{QUESTION}}
```

```
{{ANSWER}}
```

Then, we evaluated this naive MicroVQA on the top closed source models with the image, and to study the over-reliance on language shortcuts, we evaluated a text-only version. In the text-only setting, we add the following to the prompt: "If an image is mentioned, ignore this information and try your best to answer the question.". Tab. 5 shows the results of this experiment.

Table 5. Performance on MicroVQA MCQs with naive MCQ generation. We report overall VQA and VQA without the image.

	VQA	VQA-no-image
GPT-4o	85.1	82.7
Claude-3.5-Sonnet	91.4	88.4
Gemini-1.5-Pro	88.5	82.4

All models score over 80% on questions that are from a specialized domain. We hypothesize that this reflects a problem with the non-trivial task of MCQ generation from raw VQAs for several reasons. First, the high 'VQA-no-image' scores across all models suggest that selecting the correct option does not require image understanding, despite the raw VQA samples being designed to incorporate image-based reasoning. An alternative explanation is that the questions themselves may be too easy for advanced models. Second, we test this alternate explanation by performing open evaluation on a subset of the raw VQA queries– that is, we queried GPT-4o with the questions but without the options. Here, the models rarely gave good responses, even after careful prompting. This suggests that models are leveraging test-taking strategies to eliminate distractors [27]. Third, we examined the chain-of-thought responses in the VQA-no-image case, which suggested the models often eliminate distractors based on information from the text question. Therefore, we conclude that there are issues with the improper generation of the MCQs using the naive strategy.

What exactly are the problems with MCQ generation? Qualitatively, the types of errors are the same as those we discuss in Appendix E.6, so we refer readers to that section to get a deeper understanding.

E.3. Stage 1 MCQ generation (exam alignment)

The first stage transformed raw, open-ended questions and long-form answers into well-structured MCQs aligned with established principles of biomedical exam design. A physician-scientist trained in medical board exam item writing initiated the process by reviewing biomedical educational literature, NBME guidelines [64], and Bloom's taxonomy for assessing cognitive skills [5, 19, 89]. The expert manually converted 50 raw question-answer pairs into one-best-answer MCQs with biologically relevant distractors using the literature review and their prior training. This manual effort ensured that the MCQs preserved the original question's intent and scientific validity while adhering to educational standards. Distractors were crafted to require reasoning and prevent reliance on superficial cues like word association or general knowledge, thus maintaining the depth and complexity of the original content.

To scale this process, the expert designed an initial LLM prompt for automated Stage 1 MCQ generation. This prompt and the manually curated training data were input for DSPy-based prompt optimization. Using the `MIPROv2` optimizer, the prompt was refined within a Chain of Thought (CoT) framework combined with retrieval-augmented generation (RAG) pipelines powered by `o1-mini`. Optimization was guided by a custom metric evaluated by a separate LLM judge (`o1-mini`). The judge assessed each revised MCQ across three criteria: (1) semantic similarity to the original question and answer, (2) adherence to NBME formatting standards, and (3) minimization of extraneous information that could provide unintended clues. The optimization metric was weighted to prioritize preserving the original meaning and scientific validity, with lesser emphasis on formatting and extraneous text. Additional implementation details are provided in the source code.

E.4. Stage 2 MCQ generation (RefineBot) details

The goal of RefineBot is to take MCQs that are too easy for MLLMs – especially where they are leveraging language-only shortcuts – and to rewrite them to make them more challenging. It is the third panel in Fig. 4. For more details on what types of improvements are achieved, see Appendix E.6. The complete code for RefineBot is released.

A single RefineBot iteration Below, we show the pseudo-code for a single-question revision. The input is a question string, a list of options, and the index of the correct answer in the options. It returns a tuple `(code, MCQ)` where `code` is a

string on whether the run was successful, and `MCQ` is either the revised multiple choice question if the rewrite was successful or otherwise `None`.

```
def revise_mcq(question: str, choices: list, correct_idx: int, max_iters: int = 5):
   # Store original version
   original = {'question': question, 'choices': choices, 'correct_idx': correct_idx}
   reflections = []

   for iter in range(max_iters):
      # Try answering without image
      result = evaluate_without_image(question, choices, correct_idx)

      if not result.can_answer:
         code = 'SUCCESS_NO_CHANGE' if iter == 0 else 'SUCCESS_REWRITE'
         return code, (question, choices, correct_idx)

      if iter == max_iters - 1:
         return 'FAIL_ITERATIONS', None

      # Reflect on strategies used to solve
      reflection = analyze_why_answerable(result)
      reflections += reflection

      # Rewrite based on all reflections from all iterations
      new_version = rewrite_question(reflections, original)

      # Verify the rewrite didn't change the meaning meaning preserved
      if not check_meaning_preserved(original, new_version):
         return 'FAIL_REWRITE', None

      # Update current version
      question = new_version['question']
      choices = new_version['choices']
      correct_idx = new_version['correct_idx']

   return 'FAIL_UNKNOWN', None
```

This process is also described in the main text. One detail to note: the `rewrite_question` function receives the reflections from all prior iterations, not just the current one. In this way, the rewriting function can consider all possible strategies that have been identified. All LLM calls are to `gpt-4o-2024-08-06`.

To better illustrate the method, we step through a single example. This way, we can show the prompts and understand responses.

1. The evaluation of a question with the `evaluate_without_image` prompt and response:

```
Prompt
--------------------------------------------------------------------------------
The following question is supposed to be paired with an image. We will not provide the image, so answer to the
     best of your ability.
In an electron microscopy image of a mouse primary embryonic cortical neuron expressing full-length human mHTT
     (BACHD), "eye-ball" shaped features are observed in the top left corner that appear anomalous. What is the
     most likely cause of these features?
Think step by step and then output the answer in the format of "The answer is (X)" at the end.

(a) Aggregation of human mHTT protein within the neuron
(b) Ice contamination from liquid nitrogen during sample preparation
(c) Formation of intracellular vesicles during neuron development
(d) Artifacts arising from excessive heavy metal staining

--------------------------------------------------------------------------------
Response (target answer is b)
--------------------------------------------------------------------------------
To determine the cause of the "eye-ball" shaped features observed in the electron microscopy image, we can
     analyze each option step by step:

(a) Aggregation of human mHTT protein within the neuron: Aggregations of proteins can indeed appear as
     anomalous structures under electron microscopy. However, these typically have irregular shapes and may not
     consistently resemble "eye-ball" shapes.

(b) Ice contamination from liquid nitrogen during sample preparation: Ice contamination can occur during sample
      preparation, particularly if samples are not handled properly when flash-frozen. This can lead to
```

```
     anomalous features appearing in microscopy images. These features might appear as circular or irregular
     shapes, potentially looking like "eye-balls."

(c) Formation of intracellular vesicles during neuron development: Intracellular vesicles are normal structures
      within neurons and other cells. Although they can appear during development, they are typically well
     characterized and documented, and are less likely to be described as anomalous in the context given.

(d) Artifacts arising from excessive heavy metal staining: Over staining can indeed produce artifacts in
     electron microscopy images. While staining artifacts are plausible, they might not specifically result in "
     eye-ball" shapes unless the staining process consistently targets specific round structures.

Considering the nature of the descriptors ("eye-ball" shaped, anomalous), option (b) ice contamination is often
      a source of unexpected features in microscopy images that could fit the appearance described, as ice can
     form circular shapes.

Therefore, the most likely cause of these features is (b) Ice contamination from liquid nitrogen during sample
     preparation.

The answer is (b).
```

2. If the answer is incorrect, then the question is already hard enough, and we can exit. Otherwise, we call the `analyze_why_answerable` function, which reflects on how this answer was achieved. It continues in the same conversation:

```
---------------------------------------------------------------------------------
User
---------------------------------------------------------------------------------
That is correct.

Explain how you were able to answer the question without access to the image - only the question_stem and
     choices.
What strategies did you use?

Then, briefly summarize these "language-only strategies" into a short list.

Finally, say whether your reasoning was closer to:
- 'deduction': you answered with high confidence because you had enough information.
- 'abduction': you answered with lower confidence you made an informed guess.

Be concise in your final response.

---------------------------------------------------------------------------------
Assistant
---------------------------------------------------------------------------------
To answer the question without the image, I relied entirely on analyzing the given information and the
     plausible characteristics of each option. Here's a breakdown of my strategy:

1. **Keyword and Context Analysis**: I focused on key descriptors such as "eye-ball" shaped features and their
     being "anomalous" in an electron microscopy image of neurons expressing human mHTT. This guided me to
     consider characteristics commonly seen under electron microscopy.

2. **Knowledge of the Subject Matter**: I applied general knowledge about electron microscopy artifacts and
     protein behavior. This helped me weigh the likelihood of each option based on what typically causes
     specific image features.

3. **Process of Elimination**: By understanding the nature of each option-including potential artifacts or
     structures (such as vesicles or ice) in microscopy-I systematically assessed the validity of each scenario
     in relation to the described features.

4. **Logical Inference**: I inferred which scenario most commonly results in anomalies resembling "eye-ball"
     features, favoring the simplest explanation.

Summary of "language only strategies":
- Analyze keywords and context
- Apply relevant subject matter knowledge
- Use process of elimination
- Make logical inferences

The reasoning was closer to 'abduction' as I made an informed guess based on patterns and typical causes, given
      that I lacked direct visual confirmation.
```

3. Next, we call `rewrite_question`. Here, the templated variable `{{conversations}}` has the full conversations produced by the function `analyze_why_answerable` in the previous call and from all other iterations. The `{{n_chat}}` just indicates how many conversations there are included:

```
Below, I will display {{n_chat}} chat conversations between a 'user' and an LLM 'assistant'.
```

```
In each conversation
      - a user asks the assistant to answer a multichoice VQA question, however they do not provide the image.
           They only get the question_stem and choices.
      - the question_stem and correct answer are similar to other conversations, however the distractors are
           different
      - the assistant then answers correctly.
      - the user then asks the assistant to explain how it answered the question with only the text.
      - the assistant then summarizes what strategy they used to answer the question.
Altogether, these conversations give examples of language shortcuts that need to be avoided when constructing
     the question_stem and choices.

Here are the conversations:

{{conversations}}

Your task is to rewrite the question_stem and choices so that a different LLM 'assistant' cannot use the
     language-only strategies that were identified in these past conversations.
Include an 'explanation' about why your new set of distractors are better.
Your revised choices should include the correct answer at the 'correct_index'.

Your revised question_stem and choices should not significantly change the meaning of the question and correct
     answer.
You are free to change the distractors a lot to achieve this task.
Include {{n_choices}} choices.
The choices should not be made too different in length and detail to the original question stem and choices.
Note that the question stem may contain important cues that cannot be removed. E.g. if a question asks about
     the "green stain" in an image, you cannot change it to "stain" because it introduces ambiguity.
The choices should not include the letters in front like (a), (b), ...

Return a json
```

The output of this function is a JSON and is:


```
{
  "question_stem": "In an electron microscopy image of a neuron expressing human mHTT (BACHD) protein, distinct,
       spherical features are prominent and seem to be atypical. What is the most probable explanation for
      these features?",
  "choices": [
    "Concentration of cellular waste products leading to distinct morphological anomolies",
    "Formation of air bubbles introduced during sample immobilization processes",
    "Ice contamination from liquid nitrogen during sample preparation",
    "Inclusion bodies formed by aggregated proteins expressing during stress response",
    "Cellular morphological changes during mitotic division"
  ],
  "correct_index": 2,
  "explanation": "The revised options introduce plausible scientific phenomena that could also result in
      spherical or distinct anomalies. This set of distractors is more varied and grounded in legitimate
      alternative explanations without leading the responder towards the correct answer through language
      patterns. For example, air bubbles and protein inclusion bodies might mimic sphere-like structures.
      Additionally, by adding an unrelated phenomenon like cellular changes during division, the set confuses
      the language pattern strategy previously used. Thus, the correct choice of \"Ice contamination from
      liquid nitrogen\" stands out less and necessitates either visual confirmation or real understanding of
      sample preparation outcomes to identify correctly."
}
```


Notice that the response includes an explanation of why it made the changes, and the changes relate to what was in the reflection, adding interpretability.

4. Finally, we check that the rewrite has not changed the semantic meaning of the question and answer.

```
Prompt
********************************************************************************
Below are two question-answer pairs.
The question-answer pairs are part of VQA triplets, and both pairs use the same image.

Are these question-answer pairs semantically equivalent?
Or are they significantly different?
Give a true/false and also an explanation.

QUESTION 1:
In an electron microscopy image of a mouse primary embryonic cortical neuron expressing full-length human mHTT
     (BACHD), "eye-ball" shaped features are observed in the top left corner that appear anomalous. What is the
     most likely cause of these features?

ANSWER 1:
Ice contamination from liquid nitrogen during sample preparation

QUESTION 2:
```

```
In an electron microscopy image of a neuron expressing human mHTT (BACHD) protein, distinct, spherical features
      are prominent and seem to be atypical. What is the most probable explanation for these features?

ANSWER 2:
Ice contamination from liquid nitrogen during sample preparation

********************************************************************************
Response, is_equivalent: True
********************************************************************************
is_equivalent: Both question-answer pairs revolve around electron microscopy images depicting neurons
     expressing the human mHTT (BACHD) protein. The main focus in each question is on the presence of unusual
     spherical (or 'eye-ball' shaped) features. In both cases, the identical explanation is provided: ice
     contamination from liquid nitrogen during sample preparation.

While the wording differs slightly, the core components, contexts, and the ultimate explanation remain
     consistent between the two pairs. Thus, they can be considered semantically equivalent as they describe the
      same phenomenon with the same reasoning for the observed anomalies.
```

5. If the meaning check step fails, then we exit with a fail as we notice that once the meaning deviates it will become more distorted with further iterations. Otherwise, we update the MCQ, and start the loop again. Where the first function call in the new loop will check whether this revised MCQ is now too hard or needs to be rewritten.

Calibrating the preservation of meaning Since there is a risk that the rewriter will change the semantic meaning of the question and answer, it is crucial that the `check_meaning_preserved` is effective. For topics requiring domain expertise, this requires prompt engineering with a domain expert providing feedback. This is one of the key human bottlenecks in tuning the prompts in this system.

Other sources of MCQ invalidation While the `check_meaning_preserved` function checks that the question and answer is correct, an additional risk is that the rewrite introduces distractors that are more correct than the target answer. In our manual review evaluations (we reviewed all 1,042 MCQs), this was very rare. However, if this were a significant problem in future systems, it may be addressable by tuning the rewriter bot, with an expert human evaluator in the prompt engineering loop.

Extending RefineBot beyond language shortcuts We constructed RefineBot specifically to address the issue of language shortcuts – where the question is answerable without using the image. This is appropriate because we work with vision-centric VQA, meaning the questions should require image interpretation. However, the framework should work in other settings – namely language-only QA. This would require adjusting the prompts slightly. For example, the current reflection prompt asks how the question was successfully evaluated without using language; also, the current rewriter prompt directs the LLM to address language shortcuts in its rewrite. Therefore, adapting Refinebot to language-only MCQ should make these prompts more general.

On the other hand, the method could also be updated for the more general VQA setting – to improve questions that do not have language shortcuts, but are easy for some other reason. Here, the evaluator and reflector agents, which are currently LLMs, could be switched for an MLLM, which also ingests the image (though this would be expensive for current state-of-the-art models). We hypothesize that current MLLMs are so weak at image perception, that this is not necessary for MicroVQA – that simply removing language shortcuts will suffice for creating a challenging benchmark. As MLLMs improve at perceiving microscopy images, this general VQA RefineBot could become more useful for ensuring that the benchmark remains challenging.

Repeated RefineBot iterations A single run of RefineBot may exit as a FAIL, either because the rewrite changed the meaning of the question, or because the maximum number of iterations was reached. However, RefineBot can give very different outputs when changing the random seed, and we find that simply running again with a different random seed can lead to a successful run. Therefore, if the bot fails, we rerun it.

If an MCQ exited RefineBot successfully, then the evaluator LLM was fooled by the question (got the answer incorrect). Yet, the question could still be suboptimal in detectable ways: the LLM could be capable of narrowing the MCQ down to two options, but have guessed the wrong option; moreover, this question could be easier for different models. To ensure it is difficult enough overall, we require that the MCQ is answered incorrectly for two random seeds from two different models: `gpt-4o-2024-08-06` and `claude-3-5-sonnet-20241022`. If not, then it fails, and we run the RefineBot again with a different random seed.

We discuss in the experiments Sec. 5.1 how this process leads to a small evaluation bias against the models used as evaluators. That section shows that despite the bias, this process makes the MCQs generally harder for all models. Future versions of RefineBot could mitigate this by including a larger family of evaluator models.

Finally, some MCQs fail to pass the RefineBot check. In these cases, we simply keep the stage 1 MCQ.

E.5. MCQ generation results: stage 1 exam alignment

The Stage 1 pipeline produced one-best-answer MCQs that preserved the content, testing focus, and validity of the expert-generated question-answer pairs. It generated an initial set of distractors following best practices outlined in educational and NBME guidelines. A key design goal of Stage 1 was to preserve the original question content without altering or introducing new information. Secondary objectives included ensuring proper formatting according to NBME guidelines and minimizing information that might provide clues to the correct answer without reference to the image.

While the pipeline ensured alignment with these goals, preserving the original content occasionally resulted in MCQs containing extra information, such as image descriptions or specific biological terms (i.e., gene or protein name), that could inadvertently help narrow the options. For example, stage 1 question stems sometimes included protein names, disease descriptions, or descriptions of image features, which could reduce the challenge. Figure 11 illustrates Stage 1 outputs that are appropriately formatted but may preserve details from the original input, making questions less challenging. While the distractors were biologically plausible, some options were overly easy to eliminate, reducing the challenge. These limitations are addressed and refined in Stage 2.

The `o1-mini-2024-09-12` LLM judge, employed during DSPy optimization, compared the Stage 1 MCQs to the original inputs to evaluate (1) semantic similarity and (2) adherence to MCQ formatting. The results showed that 96% of questions preserved the original meaning, and 100% adhered to NBME formatting guidelines. Manual analysis of the 4% labeled as at least partially dissimilar revealed that these cases typically involved open-ended original questions being reformulated into closed-ended MCQs. This transformation, a necessary step for creating one-best-answer MCQs, was not a failure but rather an expected outcome of Stage 1. In rare instances where the original submission included multiple correct answers, the output of Stage 1 retained one correct answer and excluded others to comply with the one-best-answer format. The LLM judge flagged these cases as partially dissimilar but these changes were essential for adhering to the MCQ structure.

A common issue in MCQ design is that correct answers tend to be longer and more detailed than distractors. To assess this, we computed the ratio of tokens in incorrect options to those in the correct answer, where values ≤ 1 indicate longer correct answers, and values ≥ 1 suggest shorter correct answers. Stage 1 MCQs showed a ratio of 0.87, indicating that correct answers were only slightly longer than distractors. By comparison, naive MCQ generation yielded a ratio of 0.46, meaning correct answers were nearly twice as long as distractors, making them easier to identify. Additionally, naive MCQ generation preserved the original meaning in only 89% of cases and met NBME formatting guidelines in just 70%, highlighting the efficacy of the Stage 1 process at preserving information and ensuring proper MCQ formatting.

E.6. MCQ generation results: stage 2 RefineBot

The second stage of our MCQ generation method focuses on increasing the difficulty of the questions and options. The key idea is that we want to remove language shortcuts – information in the MCQ that allows answering the question without access to the image. To aid in understanding, we construct a toy example in Fig. 10 with the three types of language shortcuts that we identify. The questions should be paired with a stain that is mitochondria, and we want the VQA to require actually inferring that from the image. In shortcut 1, the text 'gives away' the image content so it's trivial to answer the question. In shortcut 2: The LLM generates implausible or weak distractors. In shortcut 3: 'Language bias', is a known problem in VQA [29].

Moving to real MCQs, in Fig. 4 we illustrate the transformation of a question at each stage, highlighting issues and corresponding improvements. While stage 1 produces exam-style questions that preserve the QA objective, the wording of the questions and distractors often enable MLLMs to exploit shortcuts rather than demonstrate knowledge. To address this, stage 2 (RefineBot) employs a chain-of-thought process. We analyze additional examples of improvements achieved in stage 2, showing how RefineBot improves both the style and content. The key strategies correspond to the three shortcut types that we identified:

1. (Visual giveaway) Over-description of the image eliminates the need for image interpretation. For example, in Fig. 11 (middle row), stage 1 includes details that reveal the specifics of the image, allowing the model to propose common issues without analyzing the image. Stage 2 replaces these specifics with general terms in the question stem.

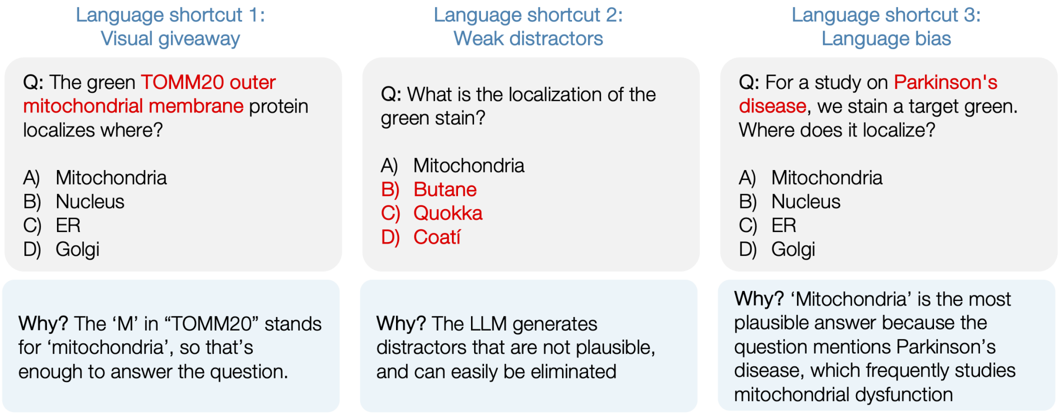

Figure 10. Three types of language shortcut relevant to MicroVQA. The target VQA has an image that is fluorescence microscopy stained with TOMM20 which would show a pattern consistent with visualizing mitochondria.

2. (Weak distractors) Distractors unrelated to the specifics of the scenario described by the question. In Fig. 11 (bottom row), stage 2 increases distractor complexity by incorporating elements tied to the question's context, making them less predictable.
3. (Language bias) Overly precise wording in the question stem: Precise phrasing can make it easy to identify the correct answer through pattern matching. For example, Fig. 11 (top row), changing 'eye-ball shaped' to 'spherical' retains the question's intent while creating more challenging distractors referencing plausible alternative processes.

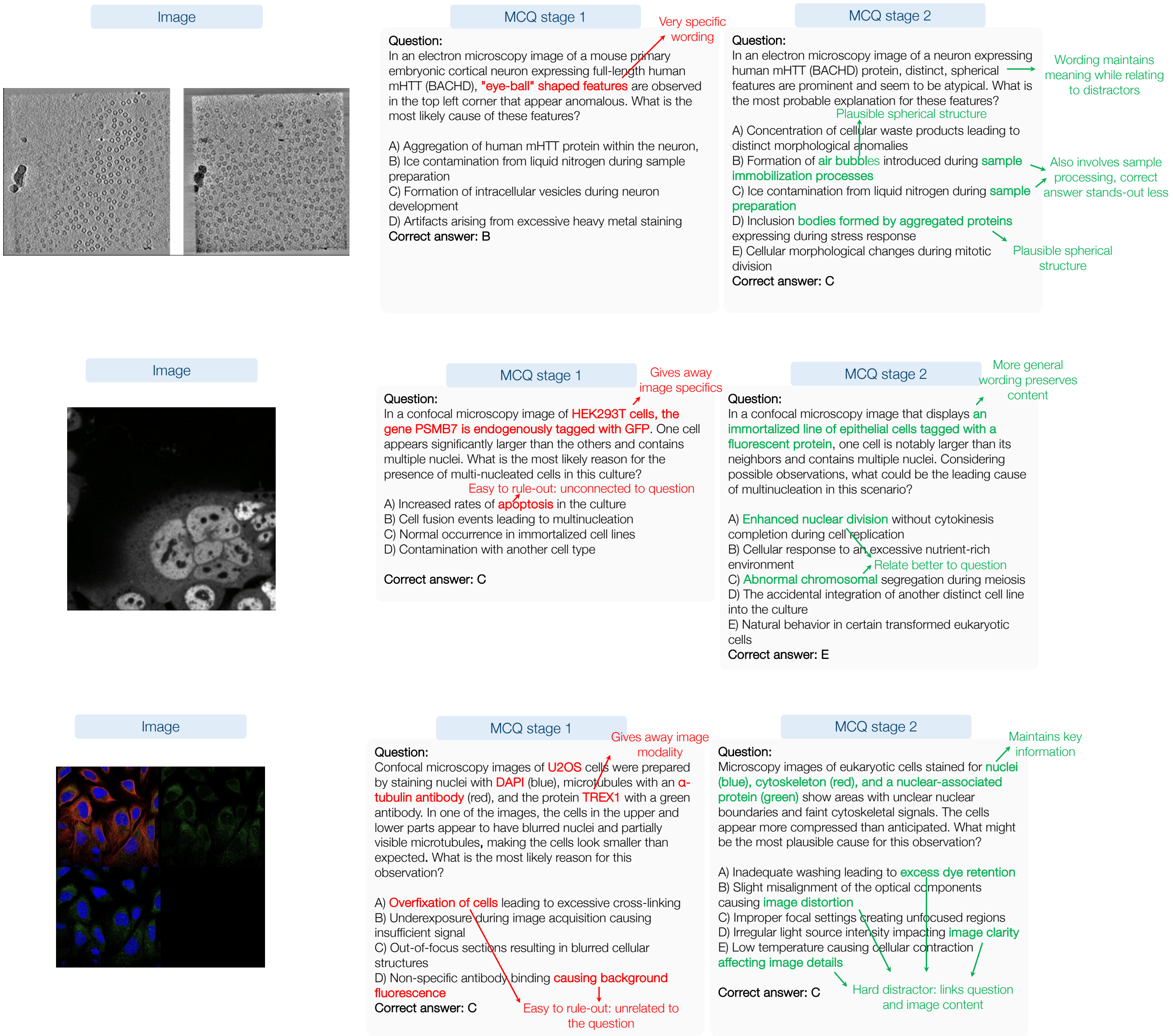

Figure 11. Examples of changes to questions and options between stage 1 and stage 2 (RefineBot) of our MCQ generation method. In red elements that need to be improved and in green improvements.

F. Experiments details

F.1. Evaluation prompts

All prompts except for LLaVA and LLaVA-Med used the chain-of-thought prompt template from the MMMU-Pro code [88]:

```
The following is a multiple choice question (with answers).
Think step by step and then output the answer in the format of \"The answer is (X)\" at the end.

{{QUESTION}}

Options:
{{CHOICES}}
```

An example complete question is:

```
The following is a multiple choice question (with answers).
Think step by step and then output the answer in the format of "The answer is (X)" at the end.

A fluorescence microscopy image displays cultured mouse primary neurons stained with a specific marker localized
    to mitochondria. The fluorescence signal exhibits an uneven distribution across the neuronal cell, and there
     is an absence of signal within the nucleus. What is the most probable cause for the uneven distribution of
    the mitochondrial signal?

Options:
  (1): Mitochondrial transport along axonal and dendritic processes ensures uneven distribution based on cellular
       energy demands.
  (2): Differential expression of mitochondrial proteins leads to variable fluorescence signal across the neuron.
  (3): Mitochondrial biogenesis occurs at specific neuronal regions, causing localized fluorescence accumulation.
  (4): Mitochondrial anchoring to the cytoskeleton restricts their movement, resulting in uneven distribution.
  (5): Mitochondrial dynamics, including fission and fusion, allow mitochondria to move and distribute throughout
       the neuron.
```

The answer is then extracted with the regex pattern: `answer is \(?([0-9])\)?`.

For LLaVA-Med, we added an extra parsing strategy. This was because we observed that while LLaVA-Med often gave reasonable responses to questions, it failed to follow instructions well. First, it did not follow the CoT prompt consistently to `think step by step`. It also didn't use the right answer formatting: `"The answer is (X)\"`. Since we were more interested in biomedical reasoning adhering to VQA formatting, we decided to handle this model differently.

Specifically, to encourage CoT reasoning, we fixed the prefix of the response to:

`Response: Let's think through this step by step and evaluate each option..`

Then we added a suffix to the end:

`After carefully considering all the options, the most likely answer is (,`

Then we continued generation, and did regular regex parsing to get the final answer.

We also find that a few models make minor but consistent errors with formatting the answer and elect to fix them. For example `x-ai/grok-2-vision-1212` commonly adds two asterisks before the answer like "The answer is **(4)**" instead of "The answer is (4)" (`GPT-4o` also did this sometimes). Some other common errors that we saw but did not handle (because it would be difficult to do rigorously) was shown by `meta-llama/llama-3.2-11b-vision-instruct`: the response would write one of the MCQ options but not state its number.

F.2. Model details

Our main benchmarking experiments in Tab. 3 refer to models that have multiple versions. The exact model versions used for inference are shown in Tab. 6

Model Name	API Endpoint	Source & Details
o1	`o1-2024-12-17`	OpenAI API
Claude-3.5-Sonnet	`*anthropic/claude-3.5-sonnet-20240620`	Openrouter API
Gemini-Pro-1.5	`google/gemini-pro-1.5`	Openrouter API
Pixtral-Large	`mistralai/pixtral-large-2411`	Openrouter API
Grok-2-Vision	`x-ai/grok-2-vision-1212`	Openrouter API
Qwen-2-vl-72b-Instruct	`qwen/qwen-2-vl-72b-instruct`	Openrouter API
VILA1.5-40b	`VILA1.5-40b`	HuggingFace, local inference
GPT-4o	`gpt-4o-2024-08-06`	OpenAI API
Llama-3.1-Nemotron-70b-Instruct	`nvidia/llama-3.1-nemotron-70b-instruct`	Openrouter API
Llama-3.2-90b-Vision-Instruct	`meta-llama/llama-3.2-90b-vision-instruct`	Openrouter API
Qwen-2-VL-7b	`qwen/qwen-2-vl-7b-instruct`	Openrouter API
Claude-3.5-Haiku	`anthropic/claude-3.5-haiku`	Openrouter API
Gemini-Flash-1.5-8b	`google/gemini-flash-1.5-8b`	Openrouter API
GPT-4o-mini	`gpt-4o-mini-2024-07-18`	OpenAI API
Pixtral-12b	`mistralai/pixtral-12b`	Openrouter API
VILA1.5-13b	`VILA1.5-13b`	HuggingFace, local inference
Llama-3.2-11b-vision-instruct	`meta-llama/llama-3.2-11b-vision-instruct`	Openrouter API
LLaVA-Med-Mistral-7B	`LLaVA-Med-Mistral-7B`	HuggingFace, local inference
LLaVA-Mistral-7B	`LLaVA-Mistral-7B`	HuggingFace, local inference

Table 6. AI model API endpoints and their sources

For LLaVA and LLaVA-med, although the version number 1.6 is different from LLaVA-Med with 1.5, we do believe that LLaVA-Med was either trained from this LLaVA version, or from one that is very similar. They have identical architecture, and the version is very close; it is possible the LLaVA-Med copied the number incorrectly. The LLaVA-Med codebase or github issues does not clarify this at the time of writing.

Note that for OpenRouter function calls to Anthropic, the date is not included in the string. We believe they use timestamps for older model versions, but not for the latest version. So reproducing it may require checking if the model has been updated.

F.3. Human baseline on MicroVQA

To perform a human baseline, we recruited co-authors who created questions. The conditions were:

- Questions were provided in a google form.
- Participants did not view any question that they created.
- Internet access was allowed, but LLM access was not. This is because realistic biological practice is knowledge-intensive, and may require looking up facts or references.
- Time limit was 3 minutes per question. They were free to take as many breaks as they wanted.
- They answered the multiple choice question without further written justification.

These conditions – 3 minutes and internet access – give sufficient time for an expert to make a reasonable attempt. Since the score was far below 100%, we claim that these questions could be of value to real experts as an LLM chat assistant. Each participant only took a subset of the questions (about 100 each). The final score is the aggregate.

F.4. More experiments on attribute-level information

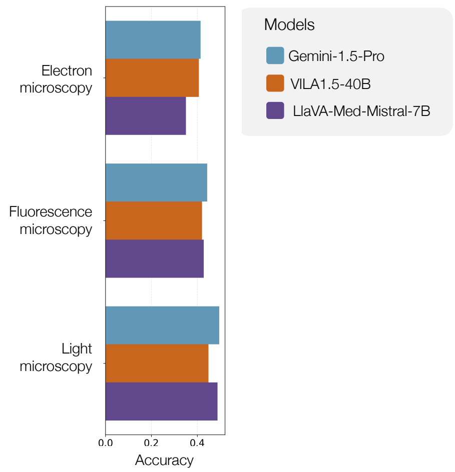

Figure 12. Performance by image modality type for the best models: Gemini-1.5-Pro (closed source), VILA1.5-40B (open-source), and LlaVA-Med-Mistral-7B (medical).

We described our metadata features in Sec. 3, and we can now leverage them to benchmark models on different attributes. Fig. 5 (left) shows all sub-tasks, and shows that 'compare image sets', which is common in biology to compare 'treatment' and 'control' groups. All models perform relatively well, despite multi-image reasoning being understudied in MLLMs [78]. Fig. 5 (right) shows that for Gemini and LLaVa-Med, higher Bloom's levels – corresponding to more advanced reasoning – leads to worse performance. This supports the intuitive result that higher-level reasoning is more challenging – although VILA does not follow this trend. A final result in Fig. 12 shows that all models performed worse on cryo-electron microscopy images than brightfield or fluorescence microscopy. This may be due to cryo-EM's challenging features (low contrast, low signal-to-noise ratio) and their under-representation in pretraining datasets [93].

F.5. Language shortcut ablations

In Tab. 4 we do language shortcut ablations for some top-performing models and some lower-performing models – both no-image and choices-only ablations.

Table 7. Ablation study on MicroVQA MCQs. Column 2 is overall accuracy. Cols 3-4 are the no-image ablation accuracy and drop compared to overall accuracy. Cols 5-6 are the choices-only ablation accuracy and drop compared to overall accuracy. See the text for discussion.

		No-image ablation		Choices-only ablation	
	Accuracy	Accuracy	Drop	Accuracy	diff
o1	52.8	49.2	-3.6	37.7	-15.1
Claude-3.5-Sonnet	51.7	46.0	-5.8	44.0	-7.7
Gemini-Pro-1.5	51.1	47.2	-3.8	36.8	-14.3
Pixtral-Large	49.8	46.3	-3.6	36.7	-13.1
Grok-2-Vision	48.4	46.3	-2.1	40.5	-7.9
GPT-4o-mini	46.2	44.2	-1.9	34.3	-11.9
Pixtral-12b	45.6	43.7	-1.9	31.8	-13.8
Random	22.0				

The no-image ablation is a common test in VQA evaluations[29, 74]. The MLLM only receives the text prompt, and we add the following sentence to the prompt template:

`If an image is mentioned ignore this information and try your best to answer the question.`

The choices-only ablation has been explored in (non-visual) question answering [7]. The model does not receive the image or the question and must guess an answer from only the choices. We add this sentence to the prompt template:

`However I will not give you the question text or the images, I will only give you the choices, so please try your best to answer the question.`

Both ablations lower the scores, but they are still above random. While this is initially surprising, it is actually consistent with other benchmarks. A key challenge in VQA benchmarks is for questions to be "vision-centric": that the question requires using the image features to answer. We still have very strong evidence that MicroVQA is vision-centric, which we discuss below.

First, let's recall the language shortcuts by considering a VQA sample with an image of a green field, with the question "what color is the grass".

- 'Language bias' is well-known for enabling models to make educated guesses about MCQs without access to the image [29]. For the question "what color is the grass?", then without the image, "green" is a likely guess, although there are other possible answers, like "brown".
- 'Visual giveaway' means there are too many image details in the question, for example if the question were "what color is the green grass?" This removes any need to look at the image.
- 'Weak distractors' gives other question choices that are easily eliminated. For example, for question "what color is the grass?", a bad distractors would be "pink". This also removes any need to look at the image.

(In Appendix E.6, we also discussed language shortcuts with biology examples as it related to MCQ construction.) Now we argue that MicroVQA is still vision-centric despite the ablation results.

1. Language bias allows cheating on vision-centric questions If answerable due to a 'language bias', the question may still require perception. A model may guess that the grass is green, but it is only a guess – the grass really could be brown [29]. Therefore correct answers under the 'no-image' ablation may still be vision-centric. On the other hand problems with 'visual giveaway' and 'weak distractor' shortcuts really do render the question not-vision-centric. The RefineBot method we employed in MCQ generation was designed to target language shortcut removal. Anecdotally, our qualitative analysis (from Appendix E.6) found that RefineBot tends to focus on fixing the visual giveaway and weak distractor shortcuts. Ideally we would perform a quantitative analysis to identify which language shortcuts are due to language bias versus the others, however analyzing these questions is very time-consuming.

2. The unsolved questions are hard because of perception. The 'no-image' ablation only tells us about the 'easy' questions, but half the quesitons are too hard for current models. Our qualitative analysis of 30 failure cases demonstrates that half of them made critical perception errors, which strongly suggests they require image understanding

In contrast to the 'vision-centric' claims from the Cambrian paper [74]: They show that many VQA benchmarks have no-image ablation scores only slightly underperforming the with-image scores, and they conclude that such VQA benchmarks "do not properly measure vision-centric capabilities". But that conclusion cannot be drawn from only the no-image ablation

(following our point 2 above). For benchmarks where MLLM's score far from 100%, the failures could mostly be due to poor image understanding. This is very plausible since visual capabilities in MLLMs are viewed as lagging behind language capabilities. Likewise, our experience with our microscopy data was that visual understanding was poor, but that language knowledge was impressive.

In point 1 above, we argued that MLLMs can use language biases to pass the no-image ablation, even if they are vision-centric questions. This is actually in agreement with the claim from Cambrian that those questions don't test vision-centric capabilities.

Finally, some non-vision centric questions are acceptable. The goal of MicroVQA is to source realistic problems from microscopy research. While this area does lean heavily on image analysis, not every question will realistically require perception, so this is acceptable.

F.6. Error analysis

Here we show samples from the manual qualitative error analysis (details in the main paper). To see all 30 examples, refer the github repo.

F.6.1 Perception errors

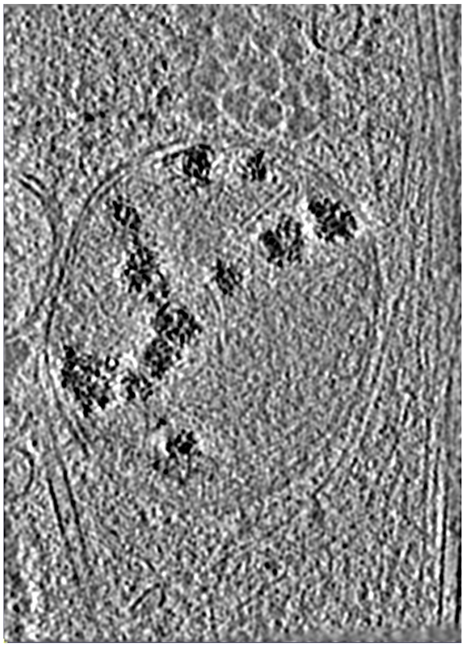

Subject: Cell and molecular biology
Bloom's level: 4 (Analysis)
Specimen: mammalian cells

Q: A cryo-electron tomography (cryoET) slice of mammalian cells was acquired at a resolution of 1.3 Å and binned by 4 (resulting in a 5.3 Å per pixel). Within the tomogram, dark circular like structures are observed in an organelle. What is the modest likely identity of these structures?

A) Ribosomes ❌
B) Phase-separated condensates such as stress granules
C) Lysosomes
D) Peroxisomes

VLM Answer: Let me think about this step by step:
1) First, let's look at what we see in the image:
- A cryo-electron tomography slice showing cellular structures
- Circular-like dark structures visible
- The resolution is quite high (5.3 Å after binning)
2) Let's consider the size and appearance of these structures:
- They appear as dense, small, roughly circular objects
- The size is consistent with ribosomes (approximately 25-30 nm)
- They show the characteristic electron density pattern of ribosomes
3) Let's analyze each option:
- Ribosomes: Match the size, shape, and density pattern
- Phase-separated condensates: Usually less defined, more amorphous
- Lysosomes: Much larger, membrane-bound organelles
- Peroxisomes: Also larger, membrane-bound organelles
4) Additional considerations:
- Ribosomes are often found associated with mitochondria
- The resolution is sufficient to resolve ribosomal structures
- The contrast and density are typical of ribosome imaging in cryoET
The answer is (1)

Error type: Perception
Error reason: These are granules characterized by heterogeneous, electron-dense aggregates. Ribosomes would be smaller, more regular in shape, and not have as high contrast.

Figure 13

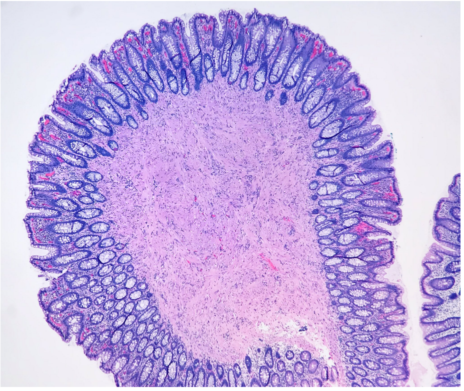

Subject: Anatomy
Bloom's level: 4 (Analysis)
Specimen: gastrointestinal lesion biopsy

Q: A hematoxylin and eosin (H&E)-stained micrograph shows cellular formations within a gastrointestinal tissue sample with unique organization and cellular patterns. What pattern and morphology do these cells most closely align with?

A) Bundles of elongated cells with pale pink cytoplasm and uniform oval nuclei in a crisscross pattern
B) Circular groups of cells with mucin in central spaces and small, dark nuclei ❌
C) Dense arrays of polygonal cells with small nucleoli and vacuolated cytoplasm
D) Rows of cells with conspicuous cytoplasm and rounded nuclei
E) Diffused arrangement of cells with transparent cytoplasm and irregularly clustered chromatin

VLM Answer: Let me analyze this step by step:
1) Looking at the H&E-stained micrograph:
- It shows a circular cross-section of gastrointestinal tissue
- There's a clear organizational pattern along the periphery
- The center shows pink-stained connective tissue (lamina propria)
2) Focusing on the cellular arrangement:
- The cells are arranged in circular groups
- These groups form glandular structures
- There are clear central spaces (lumens) in these structures
- The nuclei appear dark and are located at the base of the cells
3) Comparing with the options:
- Option 1 describes elongated cells in crisscross pattern - doesn't match
- Option 2 describes circular groups with mucin in central spaces and small, dark nuclei - matches perfectly
- Option 3 describes polygonal cells with vacuoles - doesn't match
- Option 4 describes rows of cells - doesn't match
- Option 5 describes diffused arrangement - doesn't match
4) The image shows typical intestinal glands (crypts) with:
- Circular arrangement
- Central lumens containing mucin
- Small, dark nuclei at the base of the cells
The answer is (2)

Error type: Perception
Error reason: No. The intestinal glands are not the lesion. There is a pink spindle cell lesion with a fascicular arrangement of cells

Figure 14

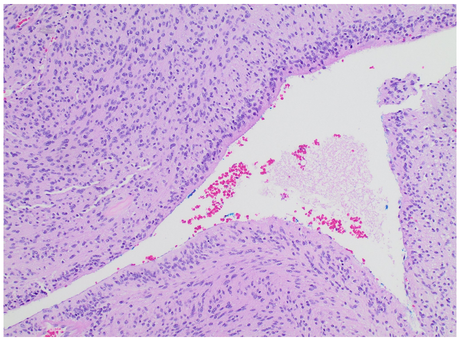

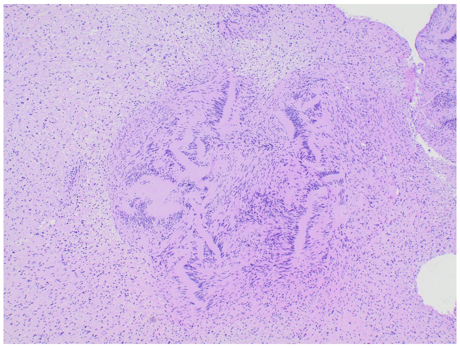

Subject: Pathology
Bloom's level: 4 (Analysis)
Specimen: schwannoma tissue

Q: An H&E-stained image of a nerve sheath tumor displays unique cell arrangements and marked changes within its structure. Given these observations, what is the most likely reason for the observed morphological alternations?

A) Indication of rapid cellular proliferation ❌
B) Sign of tumor shrinkage post-treatment
C) Result of genetic changes within the tumor cells
D) Outcome of a chronic process affecting the tumor
E) Sign of metastasis to another location

VLM Answer: Let me analyze this step by step:
1) Looking at the H&E-stained images, I can observe:
- Dense cellular proliferation
- Distinctive "herringbone" or swirling pattern
- High cellularity with numerous nuclei
- Evidence of active growth pattern
2) Key characteristics visible:
- The cells are arranged in fascicles
- There's a high nuclear to cytoplasmic ratio
- The pattern suggests active tumor growth
- No signs of necrosis or treatment effect
- No obvious signs of metastatic spread
3) Analysis of the options:
- Option 1 matches with the high cellularity and growth pattern
- Option 2 doesn't fit as there's no evidence of shrinkage
- Option 3 while possible, isn't directly evident in morphology
- Option 4 doesn't match the active growth pattern seen
- Option 5 isn't supported by the histological features shown
4) The morphological features strongly suggest active tumor growth with:
- Characteristic fascicular pattern
- High cellular density
- Well-preserved tumor architecture
- Signs of active proliferation
The answer is (1)

Error type: Perception
Error reason: No. One image shows cystic change, which can be seen in chronic, longstanding schwannomas. The other shows Verocay bodies and fascicular growth, which do not indicate rapid cellular proliferation.

Figure 15

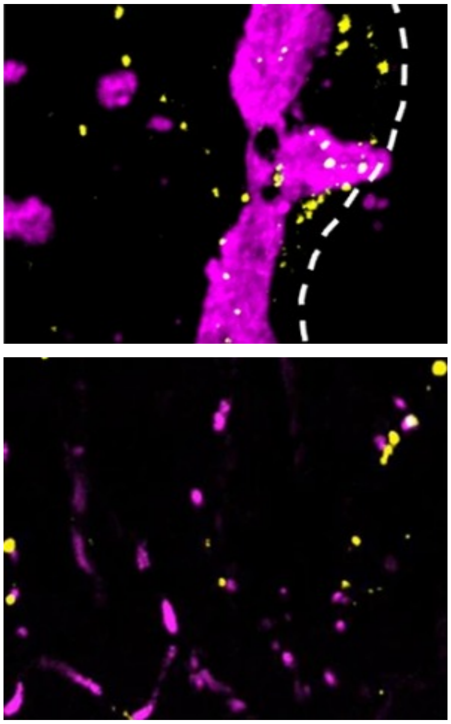

Subject: Physiology
Bloom's level: 2 (Comprehension)
Specimen: mouse liver tissue (wounded and unwounded)

Q: In a fluorescence microscopy image comparing wounded and unwounded liver tissues stained to visualize markers of immune response – neutrophil derived migrasomes (Ly6G, yellow) and platelets (CD41, magenta), what is most likely observed about the distribution of these markers in unwounded tissue?

A) Slight overlap without any notable biological consequences
B) Substantial overlap implying persistent immune activation
C) No overlap, indicating complete segregation of cellular functions ❌
D) Randomized distribution suggesting passive circulation
E) Significant separation highlighting cellular non-interaction

VLM Answer: Let me think about this step by step:
1) The image shows two types of staining:
- Yellow (Ly6G) for neutrophil-derived migrasomes
- Magenta (CD41) for platelets
2) Looking at the unwounded tissue (presumably the left image):
- There are distinct yellow dots (migrasomes)
- There are magenta structures (platelets)
- The distribution appears fairly separated
- The markers rarely overlap
3) This makes biological sense because:
- In healthy/unwounded tissue, immune cells and platelets typically maintain their distinct roles
- Without injury, there's no strong stimulus for interaction
- The cells are likely performing their normal surveillance functions
4) Analyzing the options:
- Option 1 suggests slight overlap - more than what's visible
- Option 2 suggests high overlap - not seen
- Option 3 indicates complete segregation - matches the image
- Option 4 suggests random distribution - the pattern looks more organized
- Option 5 also suggests separation - similar to option 3
5) Option 3 best matches what we observe: "No overlap, indicating complete segregation of cellular functions"
The answer is (3)

Error type: Perception
Error reason: The markers rarely overlap, but there is very focal colocalization in some puncta. The selected answer argues for "complete segregation of cellular functions", which is a too extreme or absolute conclusion based on the image.

Figure 16

F.6.2 Knowledge errors

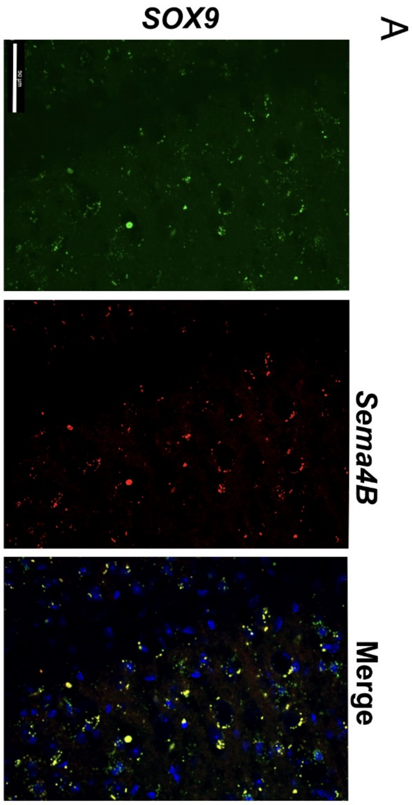

Subject: Neurobiology
Bloom's level: 3 (Application)
Specimen: Nerve cells

Q: A double immunofluorescence experiment was conducted on nerve cells, highlighting Sema4B (red marker) and Sox9, an astrocyte marker (green marker). Sema4B was found primarily surrounding the nuclear area dispersed in the cytoplasm. Based on this pattern, what potential role might Sema4B play in these cells?

A) Sema4B contributes to the regulation of metabolic pathways within the cytoplasm.
B) Sema4B is associated with intracellular trafficking processes.
C) Sema4B acts as a signaling molecule interacting with cell surface receptors.
D) Sema4B aids in transporting proteins across cell compartments. ❌
E) Sema4B is directly involved in gene transcription regulation inside the nucleus.

VLM Answer: Let me solve this step by step:
1) Looking at the immunofluorescence images:
- Sox9 (green) marks astrocytes
- Sema4B (red) shows a particular distribution pattern
- The merged image shows both markers plus nuclear staining (blue)
2) Analyzing Sema4B's distribution:
- It appears as red punctate staining
- Located primarily around nuclear areas
- Shows dispersed pattern in cytoplasm
- Not concentrated in nucleus itself
3) This distribution pattern suggests:
- The protein is involved in cytoplasmic processes
- Shows vesicular-like pattern
- Located in areas suggesting trafficking or transport
4) Looking at the options:
- Option 4 mentions protein transport
- This matches the vesicular-like pattern
- The perinuclear and cytoplasmic distribution is consistent with protein trafficking
- Other options don't align as well with the observed distribution pattern
5) Reasoning:
- The punctate pattern and distribution is characteristic of proteins involved in cellular trafficking
- The location around nucleus (where protein synthesis occurs) and throughout cytoplasm supports a transport role
- This pattern is less consistent with metabolic, signaling, or transcriptional roles
The answer is (4)

Error type: Misconception (knowledge)
Error reason: Sema4B is a transmembrane protein that plays a role in cellular signaling. TM proteins can be internalized via endocytosis and does not mean that its main function is intracellular trafficking.

Figure 17

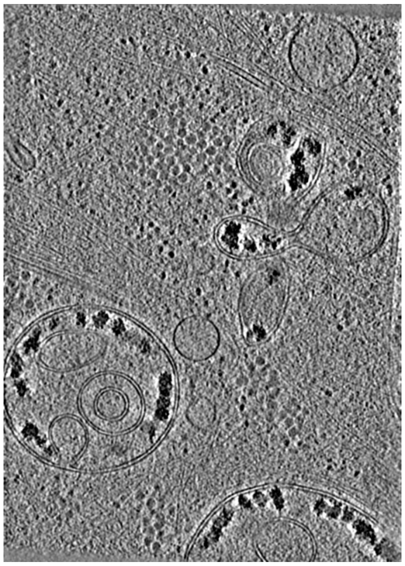

Subject: Neurobiology
Bloom's level: 4 (Analysis)
Specimen: Fibroblasts from an Alzheimer's patient

Q: A cryo-Electron Tomography image of fibroblasts from a 70-year-old patient with Alzheimer's disease shows three mitochondria gathered together. What is the most likely reason for this mitochondrial clustering.
A) Increased mitochondrial biogenesis
B) Enhanced mitochondrial fusion leading to interconnected networks
C) Mitochondrial fission due to cellular stress
D) Impaired mitophagy causing accumulation of damaged mitochondria ❌

VLM Answer: Let's solve this step by step:
1) First, let's consider the context:
- This is from a 70-year-old patient with Alzheimer's disease
- Alzheimer's is known to affect cellular function, including mitochondrial health
- We're observing clustered mitochondria in the image
2) Analyzing each option:
- Option 1 (Increased biogenesis): Would show new, healthy mitochondria, not clustering
- Option 2 (Enhanced fusion): Would show elongated, connected mitochondria
- Option 3 (Mitochondrial fission): Would show smaller, fragmented mitochondria
- Option 4 (Impaired mitophagy): Would show accumulated, potentially damaged mitochondria
3) Looking at the image:
- We see multiple mitochondria clustered together
- They appear to be intact but gathered in groups
- This suggests they're not being properly cleared from the cell
4) Considering Alzheimer's disease:
- It's known to impair cellular cleanup mechanisms
- Mitophagy is a key quality control process for mitochondria
- When impaired, damaged mitochondria accumulate instead of being removed
5) Conclusion:
The clustering is most likely due to impaired mitophagy, as this would explain why multiple mitochondria are gathering instead of being properly cleared from the cell, which is a known issue in Alzheimer's disease.
The answer is (4)

Error type: Misconception (knowledge)
Error reason: Impaired mitophagy may result in larger overall mitochondria, not an increase in small mitochondrial aggregates. Fission is a better answer in this case.

Figure 18

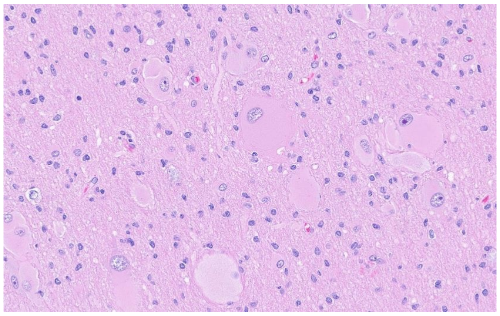

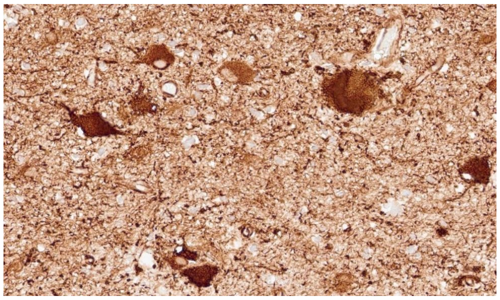

Subject: Neurobiology
Bloom’s level: 3 (Application)
Specimen: Resection specimen for epilepsy

Q: A microscopic analysis of tissue from an individual suffering from recurrent seizures involves using hematoxylin & eosin stain alongside a vimentin stain. Cells with stronger vimentin expression are significantly larger than surrounding entities. Which identity is most probable for these prominent vimentin-expressing cells?

A) Dystrophic neurons typically found around glial scars
B) Enlarged reactive astrocytes near injury sites ❌
C) Aberrant large cells characteristic of cortical malformation
D) Macrophages adapted to engulf cellular debris
E) Fibroblast proliferation in response to brain injury

VLM Answer: Let me solve this step by step:
1) First, let's analyze what we're seeing in the images:
- The H&E stain (top image) shows tissue with scattered cells and some enlarged cells
- The vimentin stain (bottom image) shows strong brown staining of certain larger cells
2) Key information from the question:
- Patient has recurrent seizures
- Vimentin-positive cells are significantly larger
- Looking for most probable identity of these cells
3) Important facts about vimentin:
- Vimentin is an intermediate filament protein
- Strongly expressed in reactive astrocytes
- Also expressed in mesenchymal cells
4) In the context of seizures:
- Seizures can cause brain injury
- Brain injury triggers astrogliosis
- Astrocytes become reactive and enlarged in response to injury
5) Looking at the options:
- Option 1: Neurons don't typically express vimentin
- Option 2: Reactive astrocytes do express vimentin and enlarge during injury
- Option 3: Cortical malformation cells wouldn't necessarily express vimentin
- Option 4: Macrophages are typically smaller
- Option 5: Fibroblasts would be rare in this context

6) The morphology and staining pattern of the large cells, combined with the clinical context of seizures, most strongly suggests reactive astrocytes.
The answer is (2) ❌

Error type: Misconception (knowledge)
Error reason: The AI is able to interpret the question and image, but lacks the knowledge to recognize the link between recurrent seizures, cortical dysplasia, and large ballooned cells.

Figure 19

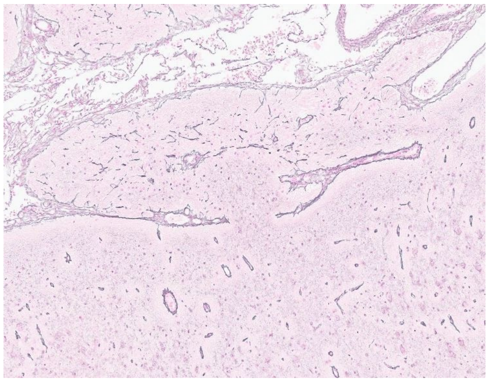

Subject: Neurobiology
Bloom's level: 4 (Analysis)
Specimen: Brain parenchyma

Q: A certain specialized stain reveals unusual cell movement crossing boundaries typical of neuronal migration in a tissue resection from a case of structural brain anomaly. Which condition is primarily associated with this type of cellular pattern?

A) Focal cortical dysplasia ❌
B) Congenital Zika syndrome
C) Schizencephaly
D) Type II lissencephaly
E) Walker-Warburg syndrome

VLM Answer: Let me solve this step by step:
1) The image shows a histological section with specialized staining that reveals abnormal cellular organization and migration patterns.
2) The question asks about unusual cell movement crossing typical neuronal migration boundaries.
3) Looking at the options:
- Focal cortical dysplasia involves disorganized neurons in localized areas
- Congenital Zika syndrome primarily affects neural progenitor cells
- Schizencephaly involves clefts in the cerebral hemispheres
- Type II lissencephaly shows abnormal neuronal migration and positioning
- Walker-Warburg syndrome is a severe form of congenital muscular dystrophy with brain malformations
4) Key characteristics of focal cortical dysplasia include:
- Abnormal neuronal migration
- Disorganized cortical layering
- Abnormal cell types crossing normal boundaries
- Disrupted cerebral cortex architecture
5) The histological image shows features consistent with focal cortical dysplasia, including:
- Disrupted cellular organization
- Abnormal migration patterns
- Cells crossing typical boundaries
The answer is (1)

Error type: Misconception (knowledge)
Error reason: Misconception about the scope and extent of neuronal migration patterns in focal cortical dysplasia (FCD) versus Type II lissencephaly. FCD will have abnormal cortical lamination, but the neurons are still within the expected gray/white matter of normal neuronal migration. They do not extend beyond and cross established neuronal boundaries such as the glial limitans.

Figure 20

F.6.3 Overgeneralization or oversimplification errors

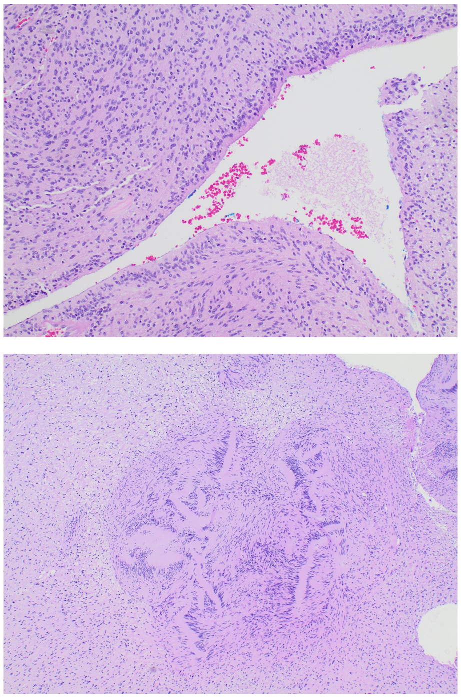

Subject: Pathology
Bloom's level: 2 (Comprehension)
Specimen: schwannoma tissue

Q: In a histological examination, an H&E-stained image shows Verocay bodies and cystic degeneration with pseudoepithelial metaplasia, indicative of schwannoma. How does the risk of malignant transformation in schwannoma compare to other peripheral nerve sheath tumors?

A) Schwannomas exhibit a lower risk of malignant transformation compared to other peripheral nerve sheath tumors.
B) Schwannomas and neurofibromas have similar risks of malignant transformation.
C) The risk of malignant transformation in schwannomas is dependent on specific genetic factors.
D) Schwannomas have an increased risk of malignancy in the presence of multiple tumors.
E) Schwannomas rarely undergo malignant transformation, regardless of tumor location or size. ❌

VLM Answer: Let me think about this step by step:
1) The images show typical histological features of schwannoma with:
- Verocay bodies (palisading nuclei)
- Cystic degeneration
- Pseudoepithelial metaplasia
2) Regarding malignant transformation in schwannomas:
- Schwannomas are typically benign tumors
- They rarely undergo malignant transformation
- This is true regardless of their size or location
- Even multiple schwannomas (as in schwannomatosis) rarely become malignant
3) Comparing to other peripheral nerve sheath tumors:
- Neurofibromas, especially plexiform types, have a higher risk of malignant transformation
- This is particularly true in the setting of NF1 (neurofibromatosis type 1)
- Malignant peripheral nerve sheath tumors (MPNSTs) more commonly arise from neurofibromas than schwannomas
4) The statement about genetic factors or increased risk with multiple tumors isn't accurate for schwannomas, though it may be true for other nerve sheath tumors
5) Looking at the options, the key is that schwannomas maintain their benign nature in most cases, regardless of circumstances.
The answer is (5)

Error type: Overgeneralization
Error reason: The selected choice does not address the question asked "compared to other peripheral nerve sheath tumors?". The "best answer" for the question should compare to other tumors as specified in the question. The AI has "oversimplified" the question to ask a simpler question than the one asked.

Figure 21

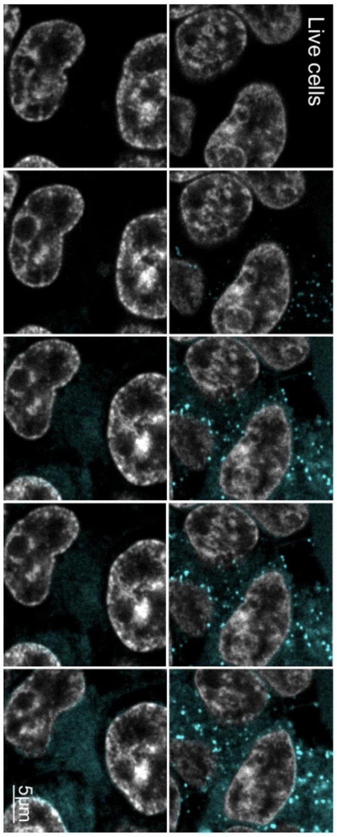

Subject: Cell and molecular biology
Bloom's level: 4 (Analysis)
Specimen: HEK293 cells (wildtype and PEX19 knockdown)

Q: Confocal microscopy images depict live HEK293 cells subjected to CRISPR/Cas9-mediated knockdown of a specific gene, alongside wildtype cells. The cells are stained with BODIPY-C12 (cyan) to highlight lipid droplets and Hoechst to stain the nuclei. In wildtype cells, distinct organellar structures are visible, whereas these structures are absent in the gene knockdown cells. What is the most likely cellular outcome resulting from the knockdown of this gene in HEK293 cells?

A) Disruption of mitochondrial fission
B) Impaired peroxisome biogenesis
C) Altered lipid metabolism pathways ❌
D) Reduced endoplasmic reticulum function
E) Decreased nuclear transport processes

VLM Answer: Let me solve this step by step:
1) First, let's analyze what we're seeing in the images:
- Live HEK293 cells are shown
- BODIPY-C12 staining (cyan) shows lipid droplets
- Hoechst staining shows nuclei (grey/white)
- The images show a time series (left to right)
2) Key observations:
- The cyan staining (BODIPY-C12) indicates the presence of lipid droplets
- There's a difference between wildtype and knockdown cells in terms of organellar structures
- The staining specifically targets lipids
3) Consider the options:
- Mitochondrial fission would primarily affect energy metabolism
- Peroxisome biogenesis affects lipid breakdown
- Lipid metabolism directly relates to lipid droplet formation
- ER function is broader than just lipid effects
- Nuclear transport isn't directly related to lipid droplets
4) Reasoning:
- Since BODIPY-C12 specifically stains lipids
- The visible differences are in lipid-containing structures
- The most direct connection would be to lipid metabolism
- Changes in lipid metabolism would directly affect lipid droplet formation and distribution
The answer is (3)

Error type: Overgeneralization
Error reason: Assumes that the changes in lipid droplets are directly linked to altered lipid metabolism pathways, without fully integrating the implications of peroxisome biogenesis in lipid metabolism and organelle dynamics.

Figure 22

F.6.4 Hallucination or errors due to added details

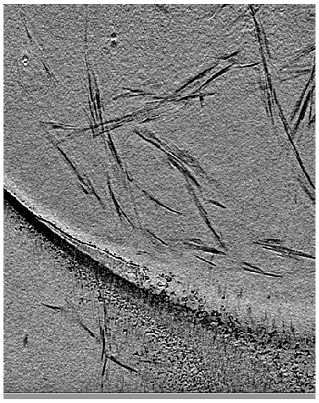

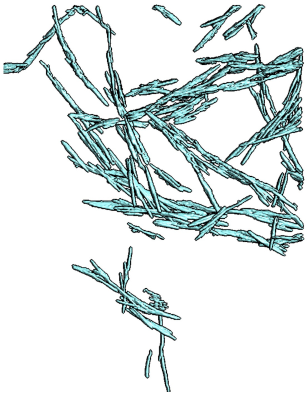

Subject: Cell and molecular biology
Bloom's level: 4 (Analysis)
Specimen: cellulose fibers in vitro

Q: A CryoET tomographic slice image and its corresponding 3D annotation of cellulose fibers in vitro show differing amounts of cellulose fibers. Beyond the 2D nature of the slice versus the 3D nature of the annotation, which mechanism most likely accounts for the observed differences?

A) Errors in the alignment during image reconstruction leading to artifacts.
B) Overlapping fibers being misinterpreted as single fibers due to resolution limits. ✗
C) Variability in manual annotation leading to subjective bias.
D) Sample dehydration causing shrinkage of fibers in the image.
E) Background noise in the image causing misidentification of fibers.

VLM Answer: Let me solve this step by step:
1) First, let's analyze what we're seeing:
- Left image: CryoET tomographic slice showing cellulose fibers in grayscale
- Right image: 3D annotation of the fibers in blue
- There appears to be a difference in how many fibers are visible/annotated
2) Consider the limitations of CryoET imaging:
- Resolution is limited
- Multiple fibers can appear to overlap in 2D views
- Depth information can be challenging to interpret
3) Evaluate each option:
- (1) Alignment errors typically cause obvious artifacts, which aren't apparent here
- (2) Resolution limits could cause overlapping fibers to appear as one in the 2D slice
- (3) Manual annotation variability wouldn't explain systematic differences
- (4) Sample dehydration would affect both views equally
- (5) Background noise might affect fiber identification but isn't the primary issue
4) The key observation is that the 2D slice shows fewer distinct fibers than the 3D annotation, likely because:
- Multiple fibers that overlap in the 2D view can be properly separated in 3D
- The resolution limits of the microscope make it difficult to distinguish overlapping fibers in the 2D view
The answer is (2)

Error type: Hallucination
Error reason: The question does not state there are systematic differences. This detail is added during the AI CoT reasoning. A better answer would rule out easy or obvious causes of differences (random or systematic). Although fibers can overlap in Cryo-EM, it is generally considered to have a reasonably high resolution.

Figure 23

F.7. Reasoning trace analysis

F.7.1 Human step-by-step reasoning

To better understand how subject matter experts approach questions in MicroVQA, we have a cell biologist and microscopist manually annotate their reasoning for each of the three main use cases.

Reasoning example for task 1 'expert visual understanding'.

```
*Question*
What subcellular structure could the bright green spots represent?*

*Answer*
This staining pattern most likely refers to cytoplasmic bodies.

*Reasoning:*
- Find green spots (no reasoning required)
- Rule out staining artefacts
    - Look at marker channels to assess whether cells appear intact
    - Compare the green spots to the marker channels for nuclei or microtubules
    - Check that green spots are always inside the cells
        - Yes, inside cells, so these are no staining artefact.
- Look up which spotty staining patterns in cells exist
    - Nuclear bodies
    - Nuclear speckles
    - Nucleoli
    - Cytoplasmic bodies
    - Centrosome
    - Vesicles
    - Intermediate filaments
    - Spotty microtubule staining
- Explore which patterns can be excluded
    - Check whether green spots are inside or outside of nuclei to know what patterns to explore
        - Outside of nuclei, thus nuclear subcellular localizations can be excluded, and cytoplasmic spotty
             patterns need to be explored
    - Check whether green spots are placed on microtubules
        - No, thus cannot be spotty microtubule stain
    - Assess localization > if accumulating around microtubule organizing center could be likely vesicles or
         centriolar satellites
        - No, not accumulating at centrosome
    - Assess size and shape of the spots
        - Small size and round, can exclude intermediate filaments
    - Count green spots per cell and check that not more than 10
        - Less likely vesicles, likely cytoplasmic bodies
```

Reasoning example for task 2 'hypothesis generation'.

```
*Question*
The sperm cells swim in circles. Wouldn't they need to swim straight to reach the egg?

*Answer*
Sperm motility and its relation to reaching the egg and fertility is very complex and still not fully understood.
      Yet, it is known that sperm swimming in circles in a shallow observation chamber like you might have
     applied, may swim on helical or twisted-ribbon structures in an in vivo or 3D space and thereby might in
     fact efficiently swim forward.

*Reasoning*
- Explore the swimming trajectories to validate what the user means with swimming in circles.
    - Detect trajectory, assess how it would be described: curved or circular swimming trajectory
- Explore what is reported on swimming trajectories of sperm cells.
    - Sperm cell swimming trajectories are species dependent
        - Validate species:
            - Assess whether the user mentioned the species in the prompt.
            - Assess look of cells in image (morphology, geometry) and compare to other images with known species
                 eventually
                - Likely human sperm.
    - Sperm cell swimming trajectories are experimental setup dependent: shallow versus deep observation chamber
        - Validate setup:
            - Brightfield microscopy, sperm cells are always swimming parallel, little to no off-focus blurring
                 of the tail.
                - Assess how images from different experimental setups look like for human sperm
                    - Likely shallow cover-slip-based observation chamber
                        - Consequences of setup for trajectory
                            In shallow observation chambers sperm cannot freely beat and thus beat parallel to
```

```
                        the glass surface, with no 3D component.
                    - Sperm cells hitting a glass surface align their beat with the glass and this way
                        can get trapped if asymmetrically beating.
                    - Curved trajectory in 2D may relate to a progressive/straight swimming path in 3D
    - Sperm cell swimming trajectories are stimulus dependent and maturation-state dependent.
        - Validate setup and explore metadata provided:
            - Check literature and compare image to available images of sperm from healthy donors (if available).
            - Compare detected swimming trajectory to swimming trajectories of sperm explored in a similar
                species and experimental setup where sperm cells are perturbed (if available).
            - Are there indications for any treatment under which the detected swimming trajectory would be
                abnormal and how would these look like (if available).
        - No indications for any treatment. Neither in prompt nor in image.
- Explore what is known on sperm cell trajectories to reach the egg in humans.
        - Not much is known since in vivo studies are not possible.
        - Sperm cells need to be able to develop hyperactive motility to swim to the egg
            - How do hyperactive sperm cells look?
                - Asymmetric beating
                    - What trajectory would they swim in in a 2D observation chamber?
                        - Curved trajectory
                            - Does that fit the swimming trajectory presented?
                                - Yes
- Assess whether when summarizing all the obtained information the conclusion from the observed swimming
     trajectory to sperm not reaching the egg is valid.
    - No.
- Compose summarizing reply to support the conclusion.
```

Reasoning example for task 3 ‘experiment proposal’.

```
*Question*
Can you suggest a simple experiment to find out whether centrosomal multiplication is occurring here?

*Answer*
One option could be to co-stain the EDC3 antibody with a centrosomal marker like PCNT or CEP250.

*Reasoning*
- Extract hypothesis to be tested from the question
    - EDC3 is labeling centrosomes and thus, centrosomal multiplication is occuring in the pictured cells
- Detect the experimental setup
    - Immunofluorescent antibody staining on a protein of interest (EDC3) in a human cultured cell type
- Compare to similar experimental setups that support such a hypothesis
    - Immunofluorescent antibody staining of centrosomes, co-labeling cell markers, performing microscopy,
        counting centrosomes
    - Acquiring or creating a transgenic cell line with a GFP-labeled centrosomal proteins, followed by
        microscopy
- Determine which methods are compatible with EDC3 staining
    - Both compatible with co-staining EDC3
- Determine which experiment is simplest for someone performing the shown experimental setup and if equal which
     one can be realized in less time
    - Immunofluorescent staining of EDC3 and centrosomes
- Find centrosome markers for human cells and assess suitability for this cell type
- Final decision on experimental setup
```

F.7.2 LLM-based error analysis

To better understand how the model approaches questions in MicroVQA, we extend our manual reasoning trace analysis from a subset of the dataset to all questions by leveraging zero-shot prompting of an LLM. The automatic error categorization achieves 63% accuracy compared to manually assigned categories. Although imperfect, automated error categorization is scalable and preserves the overall distribution of human-identified errors, enabling broader analysis. We also classify correctly answered questions to quantify overall changes across experiments. Below, we present the prompts and category definitions used for classification.

```
Below is a multiple choice question with options and the reasoning that lead a model to an incorrect response.
     Originally the model was also shown an image with the question. Your task is to use the reasoning trace to
     tag the error type:
- Perception: the image was not interpreted correctly.
- Overgeneralization: the details of the question were ignored and the general case was applied.
- Hallucination: details were added during reasoning that weren’t in the question or extracted from the image.
- Other: the error does not fit the above categories.

Question:
```

```
{{question}}

Correct answer: {{correct_answer}}

Resoning trace:
{{reasoning}}
```

```
Below is a multiple choice question with options and the reasoning that lead a model to a correct response.
     Originally the model was also shown an image with the question. Your task is to use the reasoning trace and
     determine if the question was answered because of these reasons:
- No image: the image is unnecessary because the correct answer doesn't rely on interpreting visual cues.
- Visual giveaway: the image is unnecessary because critical visual information is already described in the
     question itself.
- Language bias: the question has information that makes the correct option obvious.
- Weak distractors: the distractors are easy to rule out according to the reasoning trace.
- Good question: the question is well-constructed and needs the image and specialized knowledge to answer.
- Other: the question is hard to answer or doesn't fit the other classes.

Question:
{{question}}

Correct answer: {{correct_answer}}

Resoning trace:
{{reasoning}}
```

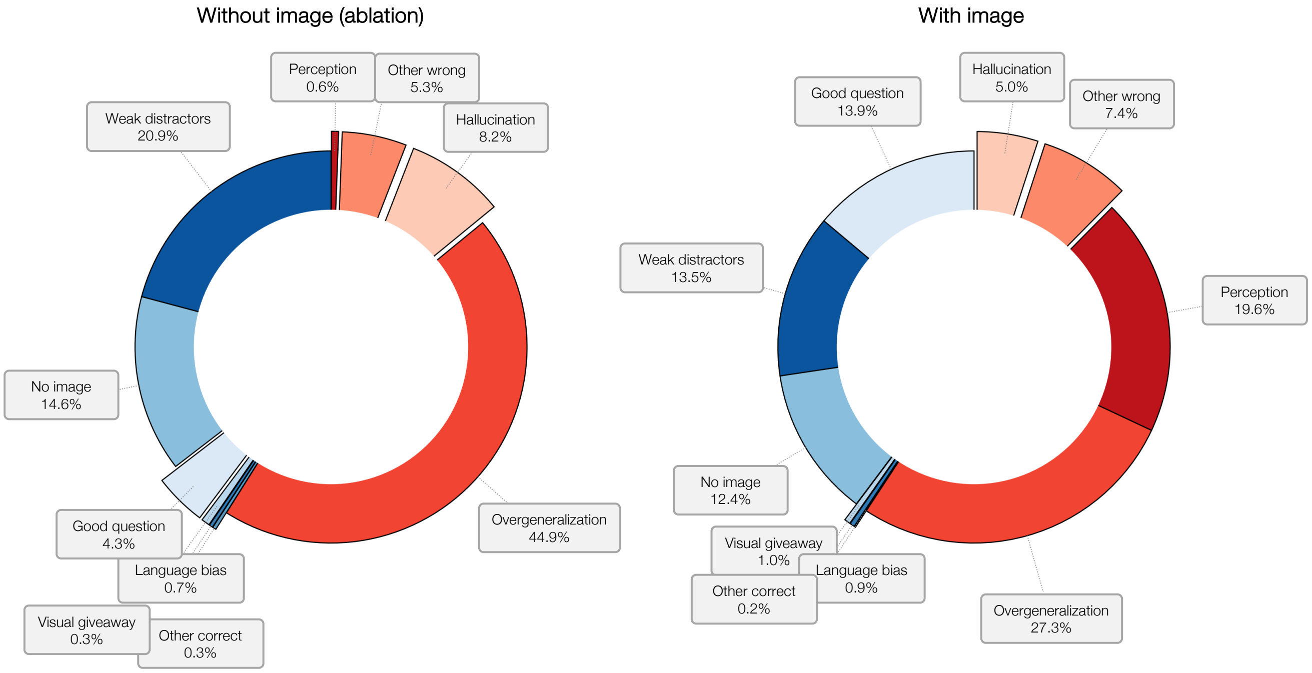

Figure 24. Reasoning trace categorization with an LLM (GPT-4o): Correct (blue) vs. incorrect (red) answers. Comparison of Claude-Sonnet's responses without (left) and with (right) image access.

We apply this system to examine the impact of providing or withholding the image for each question (Fig. 24). While overall accuracy remains comparable across both conditions, the distribution of errors and successful reasoning patterns differs meaningfully. As expected, hallucination errors decrease when the image is available, perception errors are rare when the model lacks visual input, and the proportion of questions that do not require an image remains stable.

Most errors arise from questions requiring visual and contextual understanding, highlighting the importance of multi-modal reasoning. Overgeneralization errors decrease when the image is provided, as the model is better grounded in the

question context. However, many questions remain challenging even with visual input, as perception errors increase. This suggests that effectively solving our dataset requires models to both maintain the specifics of the question's context and accurately interpret visual information.

Additionally, note that the proportion of questions answered correctly through language shortcuts (e.g., language bias and visual giveaways) remains low in both settings, suggesting the effectiveness of the RefineBot strategy. However, addressing weak distractors remains a persistent challenge for specialized datasets.

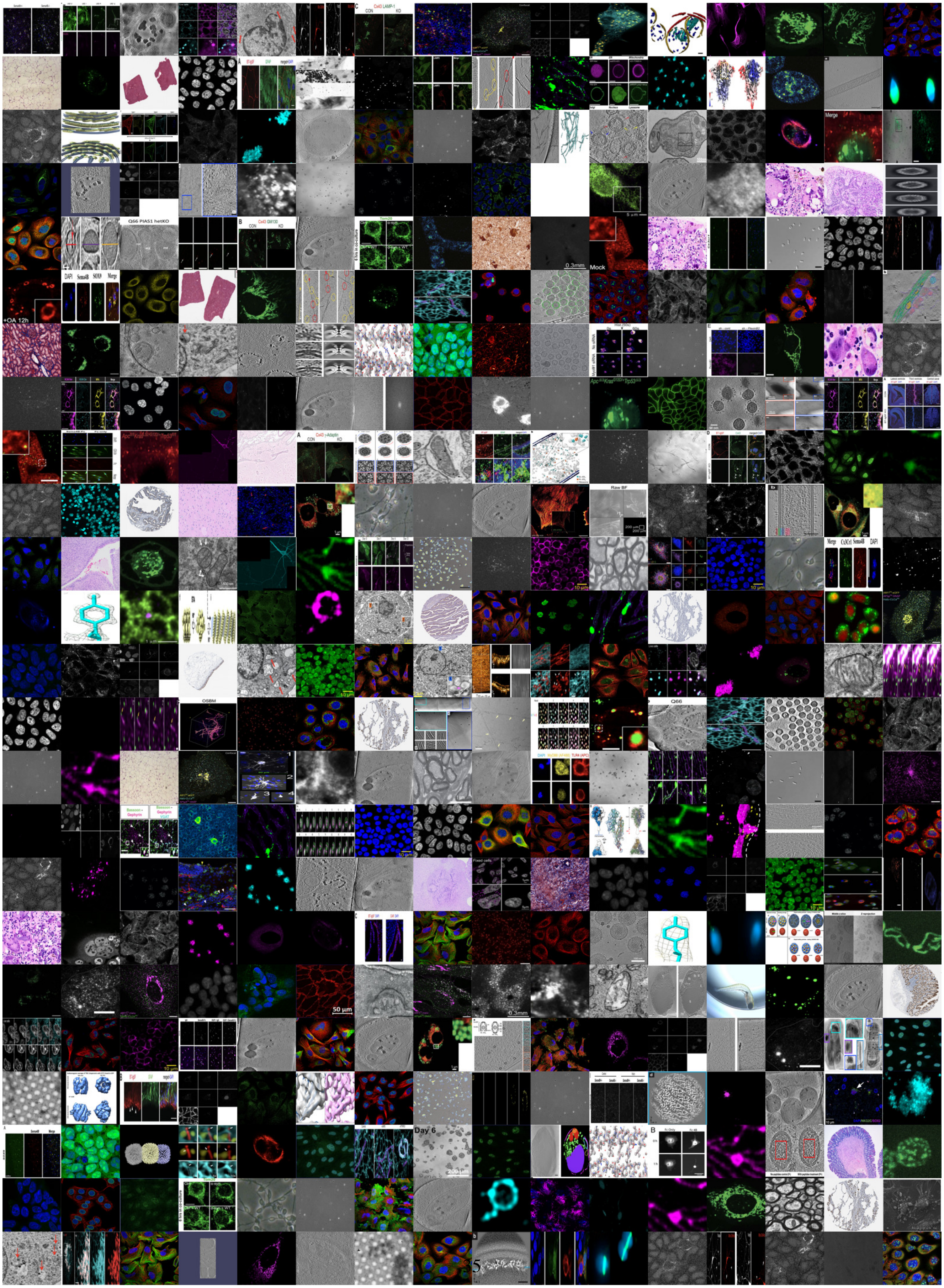

Figure 25. Collage of images from MicroVQA.
